# Effects of urinary incontinence on psychosocial outcomes in adolescence

**DOI:** 10.1007/s00787-016-0928-0

**Published:** 2016-12-10

**Authors:** Mariusz T. Grzeda, Jon Heron, Alexander von Gontard, Carol Joinson

**Affiliations:** 1 School of Social and Community Medicine, University of Bristol, Oakfield House, Oakfield Grove, Clifton, Bristol, BS8 2BN UK; 2 Department of Child and Adolescent Psychiatry, Saarland University Hospital, 66421 Homburg, Germany

**Keywords:** Psychosocial problems, Adolescence, Urinary incontinence, Cohort study, ALSPAC

## Abstract

To examine whether daytime wetting and bedwetting urinary incontinence (UI) in childhood and adolescence are associated with psychosocial problems in adolescence. We used data from the Avon Longitudinal Study of Parents and Children to examine the association between trajectories of UI from 4 to 9 years and self-reported psychosocial problems in adolescence (13–14 years) including depressive symptoms, peer victimisation, poor self-image and school experiences (negative perception of school and teachers, problems with peer relationships). Sample sizes ranged from 5162 (perception of teachers) to 5887 (self-image). We also examined associations between self-reported UI at 14 years and psychosocial problems. Relative to normative development, adolescents who experienced delayed development of bladder control had poorer self-image [standardised mean difference = 0.18 (95% CI 0.04, 0.32)], more negative perceptions of school [0.18 (0.02, 0.34)] and more problems with peer relationships at school [0.25 (0.10, 0.40)]. Persistent wetting (bedwetting with daytime wetting) in childhood was associated with increased problems with peer relationships in adolescence [0.19 (0.03, 0.34)]. The strongest associations between adolescent UI and psychosocial problems were found for daytime wetting (reference = no UI at 14 years): depressive symptoms [OR = 3.04 (95% CI 1.91–4.84)], peer victimisation [2.14 (1.48–3.10)], poor self-image (*t* = −8.49, *p* < 0.001) and problems with peer relationships (*t* = −4.69, *p* < 0.001). Children with delayed development of bladder control and persistent wetting have increased psychosocial problems in adolescence. Adolescents with UI reported a range of psychosocial problems and clinicians should be aware that they might require support from psychological services.

**Electronic supplementary material:**

The online version of this article (doi:10.1007/s00787-016-0928-0) contains supplementary material, which is available to authorized users.

## Introduction

Bedwetting (enuresis) and daytime wetting are common paediatric problems. Most children attain daytime bladder control at around 3 years [1] and nighttime bladder control between the ages of 4 and 6 years [2], but it is not uncommon for school-age children to experience urinary incontinence (UI) [3, 4]. In a large UK cohort Avon Longitudinal Study of Parents and Children (ALSPAC), parents of 7½ year-old children reported that 15.5% experienced some level of bedwetting [5] and 7.8% experienced daytime wetting [6] (comprising children with bedwetting or daytime wetting occurring ‘less than once a week’; ‘about once a week’; ‘2–5 times a week’; ‘nearly every day; or ‘more than once a day’). Among the children in the ALSPAC cohort, at 7½ years 2.6% were wetting the bed twice or more per week and 1.0% experienced daytime wetting at this frequency.

UI in school-age children is associated with emotional distress, behaviour problems and lower quality of life in cross-sectional studies [6–10] and poor self-image in a small case–control study [11]. It is believed that the association between psychosocial problems and incontinence is bidirectional. Whilst behaviour problems and difficult temperament in early childhood have been linked to an increased risk of later incontinence [12], psychological distress may also emerge when children become aware that UI is unusual for their age, or in the face of negative reactions from their parents or peers. A prospective study design is needed to examine whether childhood incontinence is a risk factor for later psychosocial problems. Only two cohort studies have examined this, with one reporting an association between enuresis (defined as involuntary voiding of urine by day or night at least once per month) and behaviour problems and mental health disorders at 11 and 13 years [13] and another reporting that children with bedwetting after age 10 had increased behaviour problems and anxiety/withdrawal up to age 15, compared with children who ceased bedwetting before age five [14]. These earlier studies used both parental and self-reports of psychological problems. As children grow older, urinary incontinence is seen as more of a burden [15] and levels of parental intolerance increase [16]. This may cause parents to be more negatively predisposed to their children and, hence, more likely to report psychological problems.

These earlier cohort studies did not distinguish between children who had bedwetting with or without daytime wetting, a potentially important omission given the evidence that children with combined (day and night) wetting have more psychological problems than children with bedwetting alone [7, 10, 17, 18]. However, no studies have prospectively examined whether children who suffer from combined wetting have poorer psychosocial outcomes in adolescence compared with bedwetting alone. Studies have described different patterns (longitudinal phenotypes) of typical and atypical development of bladder control during childhood. These include distinct developmental trajectories of bedwetting [19–21] and daytime wetting [22] and parallel trajectories of bedwetting and daytime wetting [23]. It is not known whether these trajectories are differentially associated with psychosocial problems in adolescence.

We prospectively examined whether trajectories of daytime wetting and bedwetting in childhood are differentially associated with a range of psychosocial problems in adolescence including depressive symptoms, peer victimisation, self-image and school-related outcomes. We also examined whether adolescents with current UI have increased levels of psychosocial problems.

## Methods

### Participants

The sample comprised participants from the Avon Longitudinal Study of Parents and Children. Detailed information about ALSPAC is available on the study website (http://www.bristol.ac.uk/alspac), which includes a fully searchable dictionary of available data (http://www.bris.ac.uk/alspac/researchers/data-access/data-dictionary). Pregnant women resident in the former Avon Health Authority in south-west England, having an estimated date of delivery between 1/4/91 and 31/12/92, were invited to take part, resulting in a cohort of 14,541 pregnancies and 13,973 singletons/twins (7217 boys and 6756 girls) alive at 12 months [24]. Ethical approval for the study was obtained from the ALSPAC Law and Ethics committee and local research ethics committees.

### Exposure: latent classes of daytime wetting and bedwetting in childhood

At ages 4½, 5½, 6½, 7½ and 9½ years (hereafter referred to as 4–9 years) parents were asked “How often does your child wet him/herself during the day?” and “during the night?” and for both questions were given the response options ‘Never’; ‘Occasional accidents but less than once a week’; ‘About once a week’; ‘2–5 times a week’; ‘Nearly every day; and ‘More than once a day’. An earlier study used this data to extract five trajectories (latent classes) of UI during childhood using longitudinal Latent Class Analysis (LLCA) [23]. The normative class had the highest prevalence (63.0% of the sample) and was characterised by a very low probability of daytime wetting or bedwetting by age 4. There were four atypical classes describing different patterns of childhood UI: delayed attainment of bladder control (decreasing probability of bedwetting from 6 to 9 years with accompanying daytime wetting—8.6% of the sample); bedwetting alone (15.6%); daytime wetting alone (5.8%) and persistent wetting (persistent bedwetting to age 9 with accompanying daytime wetting) (7.0%). Details of the analysis employed to extract the latent classes are provided in Online Resource Part 1. Girls had higher odds of membership in the daytime wetting class [multinomial odds ratio = 1.80 (95% CI 1.40, 2.31)] and lower odds of membership in the classes characterised by delayed bladder control [0.81 (0.69, 0.94)], bedwetting alone [0.42 (0.37, 0.48)] and persistent wetting [0.53 (0.44, 0.64)].

### Self-reported binary outcomes

*Depressive symptoms* Study children completed the Short Mood and Feelings Questionnaire (SMFQ) [25] at 13 years and 10 months (SD 0.21, range 12.5–15.2 years; hereafter referred to as 14 years). We defined high levels of depressive symptoms as scores at or above 11. This cut off has been shown to have a high sensitivity and specificity [26, 27].

*Peer victimisation* We assessed peer victimisation at 12 years 10 months (hereafter referred to as 13 years) using a modified version of the Bullying and Friendship Interview Schedule [28]. Participants were asked whether they had experienced nine different types of peer victimisation in the past 6 months, responding “no,” “yes sometimes” (<4 times), “yes repeatedly” (≥4 times), or “yes very frequently” (at least once per week). The items related to both relational and overt victimisation. We used an overall measure of peer victimisation created from the sum of all questions relating to victimisation (children scored 0 if they had never been bullied). The range of scores was 0–25 (mean 1.85, SD 2.78) with Cronbach’s *α* of 0.73 and we dichotomized the scale, defining frequent victimisation by scores at or above 4 [28].

### Self-reported continuous outcomes

*Self*-*image* The Self-Image Profile assesses attributes by which children identify themselves [29]. Participants were asked at 13 years 10 months (hereafter referred to as 14 years) to rate themselves against a list of 25 attributes (e.g. “kind”, “friendly”, “different from others”, “worries a lot”). Responses were always, mostly, sometimes, not often or never and were scored as 1, 2, 3, 4 and 5, respectively.

*School experiences* A self-completion postal questionnaire, sent to study participants at 14 years, comprised 39 questions about school-related experiences [30]. The questions were in the form of statements such as ‘my school is a place where: “I feel happy”, “other pupils accept me”, “teachers and pupils trust each other”. Responses were strongly agree, agree, disagree or strongly disagree and were scored 1, 2, 3 and 4, respectively.

No published cut-offs exist for the continuous outcome variables described above. We therefore derived continuous scales using Rasch modelling (see Online Resource Part 2).

### Confounders

We adjusted for gender and developmental level, assessed at 18 months using a questionnaire developed by ALSPAC including items from the Denver Developmental Screening Test [31] and comprising four domains of development (fine motor, gross motor, communication and social skills). Scores on each domain were adjusted for age in weeks, standardised and reversed where appropriate so that high values on all scores reflected a lower level of development. We adjusted for a total development score derived from the sum of the scores on each domain.

Early psychological problems were assessed at 3½ years using the Revised Rutter Parent Scale for Preschool Children [32]. The questionnaire comprises 43 statements describing behaviours and mothers are asked to rate the extent to which each statement describes their child on a scale comprising the options 1 (certainly true), 2 (sometimes true) and 3 (not true). Responses were aggregated to create scores in four domains: emotional difficulties, conduct difficulties, hyperactivity and prosocial behaviour. High scores indicate more problems (except the prosocial behaviour scale where low scores indicate more problems).

Binary variables for socioeconomic position and adversity were derived from responses to a questionnaire, completed by mothers during the antenatal period. Social class was dichotomized into non-manual (professional, managerial or skilled professions) and manual (partly or unskilled occupations) (1991 British Office of Population and Census Statistics OPCS); early parenthood (<19 years versus ≥19 years), housing adequacy (yes/no), maternal education (none versus high school qualifications or greater), major financial difficulties (yes/no), family size (<3 children versus ≥3 children) and the presence of a social support network (yes/no).

### Statistical analysis

*Univariable and multivariable regression* We used the latent classes described above as the exposure in our analysis. We examined the association between the latent classes and the binary outcomes using logistic regression and the standardised continuous outcomes using linear regression. Odds ratios/mean differences (as appropriate) were estimated with reference to the “normative” class. Parameter estimates were then adjusted for the confounders. All regression models were estimated using Latent Gold (version 5.0).

*Bias*-*adjustment* For the logistic regression, we obtained parameter estimates using the “Modal ML” 3-step method [33]. This enabled outcome data to be incorporated whilst avoiding any distortion of the latent class solution. In step one, the latent class model is estimated using an unconditional LLCA (i.e. a model in the absence of covariates). This model is used to derive class-assignment probabilities (i.e. the probability of membership to each class). Respondents are then assigned to the class for which their probability is greatest creating a non-latent classification (step two). Finally in step 3, measurement error inherent in the non-latent classification is quantified and used to reproduce latent classes using a set of logit constraints. This approach has been shown to produce less-biassed estimates than traditional three-step methods such as standard probability weighting or modal assignment, whilst avoiding the problem of covariates impacting on the measurement model itself. For the linear regression models, the “Modal BCH” three-step routine was employed. This achieves the same objective as Modal ML but has recently been shown to be the preferred model for continuous outcome variables [34] since even with a three-step approach it is possible for the latent class solution to be distorted by the distribution of the dependent variable.

*Missing data* We conducted the analyses on the sample (*n* = 8751) with valid data on urinary incontinence for at least three out of five measurements conducted when children were aged 4–9 years. We have previously shown our findings to be robust to the sample chosen [21], and there is little benefit to including those providing a small amount of childhood data since few provide follow-up data in adolescence. Of the 8751 participants, valid data for psychosocial outcomes were available for 5578 (for victimisation) 5631 (depressive symptoms) 5887 (self-image) 5171 (perception of school) 5169 (peer relations at school) and 5162 (perception of teachers).

*Sensitivity analysis* As a sensitivity analysis, models were re-estimated following the exclusion of participants with current bedwetting and/or daytime wetting in adolescence. This was determined by a self-report questionnaire sent out to study children when they were 14 years old asking: “Many of us have accidents sometimes. How often do the following happen to you?”: “Wet the bed at night” and “Wet yourself during the day”. The following options were given: “Never”, “Occasionally, but less than once a week”, “About once a week”, “2–5 times a week”, “Nearly every day” and “More than once a day”. This sensitivity analysis was done to examine whether childhood incontinence is associated with psychosocial problems in adolescents whose childhood continence problems have ceased. At 14 years, daytime wetting was reported by 2.9% (1.3% males and 4.2% females, *p* < 0.001) and bedwetting was reported by 2.5% (2.9% males and 2.2% females, *p* = 0.08). These analyses are not mentioned further since this exclusion had a negligible impact on the results (see Online Resource Part 3: tables S1 and S2 and figures S1 and S2).

### Association between adolescent incontinence and psychosocial outcomes

We examined whether adolescents with daytime wetting and/or bedwetting had more psychosocial problems than those with no urinary incontinence in adolescence. Urinary incontinence in adolescence was determined by a self-report postal questionnaire completed at median age 13 years 10 months (hereafter referred to as 14 years) [23]. We examined these associations using logistic regression for the binary outcomes and t tests for the continuous outcomes. The reference group was no UI (daytime wetting or bedwetting) at age 14.

## Results

Depressive symptoms were reported by 6.8% of the sample at 14 years and were more prevalent among girls (girls = 8.7%, boys = 4.9%, *p* < 0.001). Peer victimisation was reported by 17.8% of the sample (girls = 18.3%, boys = 17.2%, *p* = 0.255). Girls had poorer self-image than boys [standardised mean difference = 0.49 (95% CI 0.44, 0.54), *p* < 0.001], but boys had more negative perceptions of school [−0.07 (−0.13, −0.02), *p* = 0.008] and more problems with peer relationships [−0.09 (−0.14, −0.03), *p* = 0.002]. There was no gender difference with regard to perception of teachers [0.005 (−0.05, 0.06), *p* = 0.860].

### Associations between the latent classes and adolescent psychosocial outcomes

**Fig. 1:**
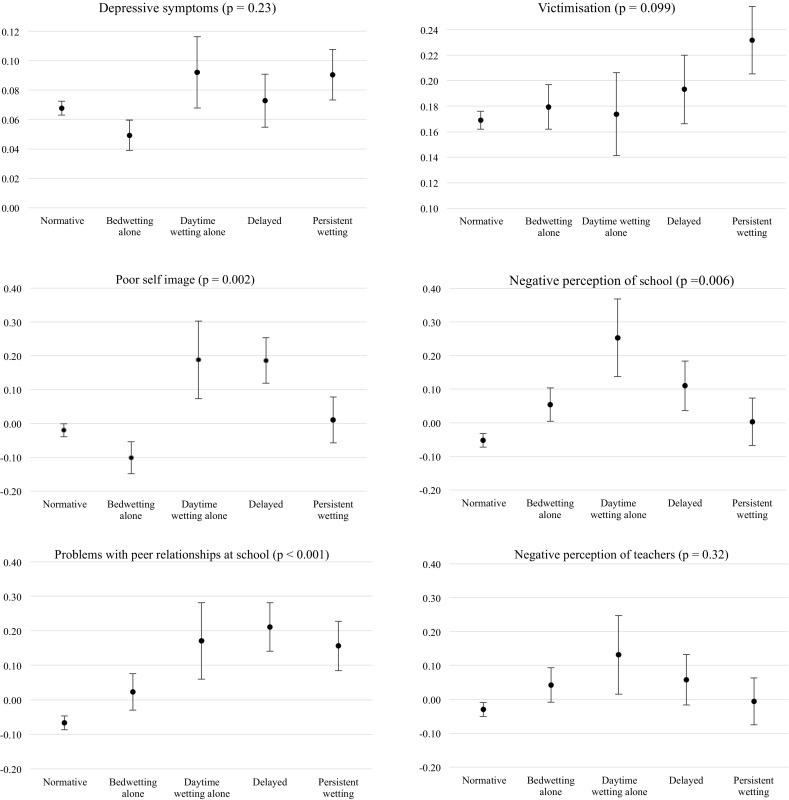
Estimated mean differences for continuous outcomes and prevalence of each binary outcome across the latent classes of daytime wetting and bedwetting at 4–9 years. For (i) and (ii) estimates shown are predicted probabilities of a positive outcome from the logistic model with 1 SE *error bars*. For (iii) and (vi) estimates are within-class means on the standardised Rasch scales, with 1 SE *error bars*

Table 1 provides the odds ratios and 95% confidence intervals for the associations between the latent classes and the binary outcomes and Table 2 provides the mean differences (expressed in standard deviations) and 95% confidence intervals for the continuous outcomes for each latent class relative to the normative class. Figure 1 shows the estimated mean differences for the continuous outcomes and the prevalence of each binary outcome across the latent classes of daytime wetting and bedwetting at 4–9 years.

**Table 1:** Odds ratios and 95% confidence intervals for the association between the latent classes of daytime wetting and bedwetting at 4–9 years and victimisation and depressive symptoms

	Unadjusted	Adjusted 1	Adjusted 2
	OR (95% CI)	OR (95% CI)	OR (95% CI)
*Depressive symptoms*			
*n*	5631	5268	4865
Normative	0.0 ref	0.0 ref	0.0 ref
Bedwetting alone	0.72 (0.45, 1.15)	0.96 (0.61, 1.52)	0.96 (0.59, 1.54)
Daytime wetting alone	1.40 (0.75, 2.59)	1.22 (0.62, 2.39)	1.09 (0.52, 2.27)
Delayed	1.08 (0.63, 1.86)	1.04 (0.58, 1.88)	1.00 (0.54, 1.86)
Persistent wetting	1.37 (0.89, 2.11)	1.52 (0.96, 2.42)	1.42 (0.88, 2.29)
	*p* = 0.230	*p* = 0.480	*p* = 0.700
*Victimisation*			
*n*	5578	5217	4814
Normative	0.0 ref	0.0 ref	0.0 ref
Bedwetting alone	1.07 (0.83, 1.40)	1.12 (0.85, 1.47)	1.11 (0.84, 1.47)
Daytime wetting alone	1.03 (0.65, 1.66)	0.96 (0.58, 1.59)	0.92 (0.54, 1.55)
Delayed	1.18 (0.83, 1.67)	1.08 (0.74, 1.59)	1.01 (0.68, 1.50)
Persistent wetting	1.48 (1.09, 2.01)	1.36 (0.99, 1.89)	1.30 (0.93, 1.81)
	*p* = 0.099	*p* = 0.330	*p* = 0.530

*Adjusted 1* adjusted for gender and socioeconomic variables, *Adjusted 2* further adjusted for developmental level and early psychological problems

**Table 2:** Mean differences (and 95% confidence intervals) for the continuous outcomes for each latent class relative to the normative class

	Unadjusted	Adjusted 1	Adjusted 2
	Difference (95% CI)	Difference (95% CI)	Difference (95% CI)
*Poor self*-*image*			
*n*	5887	5520	5097
Normative	0.0 ref	0.0 ref	0.0 ref
Bedwetting alone	−0.08 (−0.19, 0.03)	0.07 (−0.04, 0.18)	0.07 (−0.07, 0.22)
Daytime wetting alone	0.21 (−0.03, 0.45)	0.13 (−0.12, 0.38)	0.12 (−0.12, 0.36)
Delayed	0.21 (0.07, 0.34)	0.23 (0.09, 0.36)	0.18 (0.04, 0.32)
Persistent wetting	0.03 (−0.11, 0.17)	0.10 (−0.04, 0.24)	0.06 (−0.08, 0.20)
	*p* = 0.002	*p* = 0.0019	*p* = 0.035
*Negative perceptions of school*			
*n*	5171	4862	4502
Normative	0.0 ref	0.0 ref	0.0 ref
Bedwetting alone	0.11 (−0.01, 0.22)	0.09 (−0.02, 0.21)	0.07 (−0.07, 0.21)
Daytime wetting alone	0.30 (0.06, 0.55)	0.23 (0.01, 0.46)	0.23 (−0.22, 0.67)
Delayed	0.16 (0.02, 0.31)	0.20 (0.05, 0.35)	0.18 (0.02, 0.34)
Persistent wetting	0.06 (−0.09, 0.20)	−0.01 (−0.16, 0.14)	0.00 (−0.15, 0.16)
	*p* = 0.006	*p* = 0.006	*p* = 0.033
*Problems with peer relationships at school*			
*n*	5169	4858	4499
Normative	0.0 ref	0.0 ref	0.0 ref
Bedwetting alone	0.09 (−0.03, 0.21)	0.07 (−0.05, 0.19)	0.07 (−0.07, 0.20)
Daytime wetting alone	0.24 (0.01, 0.47)	0.23 (−0.01, 0.47)	0.23 (−0.22, 0.67)
Delayed	0.28 (0.14, 0.42)	0.27 (0.13, 0.42)	0.25 (0.10, 0.40)
Persistent wetting	0.22 (0.08, 0.37)	0.19 (0.04, 0.34)	0.19 (0.03, 0.34)
	*p* < 0.001	*p* < 0.001	*p* < 0.001
*Negative perception of teachers*			
*n*	5162	4853	4493
Normative	0.0 ref	0.0 ref	0.0 ref
Bedwetting alone	0.07 (−0.04, 0.19)	0.08 (−0.04, 0.20)	0.06 (−0.06, 0.18)
Daytime wetting alone	0.16 (−0.08, 0.40)	0.08 (−0.17, 0.32)	0.10 (−0.10, 0.31)
Delayed	0.09 (−0.06, 0.24)	0.13 (−0.03, 0.28)	0.13 (−0.03, 0.29)
Persistent wetting	0.02 (−0.12, 0.17)	0.00 (−0.15, 0.14)	0.01 (−0.14, 0.16)
	*p* = 0.320	*p* = 0.260	*p* = 0.350

*Adjusted 1* adjusted for gender and socioeconomic variables, *Adjusted 2* further adjusted for developmental level and early psychological problems

For the binary outcomes, there was evidence in the unadjusted model that the odds of victimisation were increased among members of the persistent wetting class relative to the normative class (Table 1), but this association was attenuated in the adjusted models. There was no evidence for differences in levels of depressive symptoms in the atypical classes compared with the normative class.

For the continuous outcomes, members of the delayed class had poorer self-image, more negative perceptions of school and a higher level of problems with peer relationships at school than those with normative development of bladder control (Table 2). These associations remained after adjustment for confounders. Children with daytime wetting alone had more negative perceptions of school and more problems with peer relationships in the unadjusted models, but these associations were attenuated in the adjusted models. Persistent wetting (persistent bedwetting with daytime wetting) was associated with increased problems with peer relationships at school and this association remained in the adjusted model. Children in the atypical classes did not have more negative perceptions of teachers compared with those in the normative class. It is notable that children with ‘bedwetting alone’ did not have higher levels of any of the psychosocial problems in adolescence compared to those with normative development of bladder control.

### Association between urinary incontinence in adolescence and the psychosocial outcomes

At age 14, daytime wetting was reported by 2.9% (167/5803) (1.5% with previous daytime wetting at 4–9 years and 1.4% with new onset) and bedwetting was reported by 2.5% (144/5805) (1.8% with previous bedwetting at 4–9 years and 0.65% with new onset).

Compared to those with no UI (no daytime wetting or bedwetting) at age 14, daytime wetting at 14 years was associated with increased levels of depressive symptoms [OR = 3.04 (95% CI 1.91–4.84)], peer victimisation [OR = 2.14 (1.48–3.10)], poor self-image (*t* = −8.49, *p* < 0.001) and problems with peer relationships (*t* = −4.69, *p* < 0.001). Bedwetting at 14 years was associated with increased levels of peer victimisation [OR = 2.50 (1.66–3.75)] and poor self-image (*t* = −3.86, *p* < 0.001). There were increased levels of depressive symptoms in females with bedwetting [OR = 2.29 (1.06–4.95)], but not males [OR = 1.20 (0.37–3.90)]. Adolescents with combined daytime wetting and bedwetting had poorer self-image than those with no UI (*t* = −2.35, *p* = 0.024).

## Discussion

### Summary of main findings

Studies of adolescent psychosocial outcomes associated with childhood incontinence are scarce and none have examined outcomes associated with developmental trajectories of UI. This study is the first to examine whether different trajectories of UI in childhood are differentially associated with psychosocial problems in adolescence. We find evidence that adolescents who experienced delayed development of bladder control have poorer self-image, more negative perceptions of school and more problems with peer relationships at school than those with normal development. Those who experienced persistent bedwetting with daytime wetting in childhood had increased problems with peer relationships in adolescence. There was strong evidence that adolescents with current UI had higher levels of psychosocial problems compared to those with no UI. The most adverse outcomes were found for daytime wetting, with increased levels of depressive symptoms, peer victimisation, poor self-image and problems with peer relationships at school. Adolescents who suffered from bedwetting reported more peer victimisation, poorer self-image and depressive symptoms (females only) and those with combined wetting had poorer self-image.

### Strengths and limitations

Our study is based on a large birth cohort and we were able to take advantage of previously derived latent classes of UI in childhood (derived from repeated measures of daytime wetting and bedwetting from 4 to 9 years) and data on a range of self-reported psychosocial outcomes in adolescence. Earlier cohort studies examining the association between childhood UI and adolescent outcomes have used parent and child reports of psychopathology and behaviour problems [13, 14]. We focussed on self-reports due to concerns that parental reports may be biassed. With the exception of depressive symptoms, there was no other self-reported data on psychopathology or behaviour problems in adolescence.

This study found no evidence that children with either daytime wetting or bedwetting alone have increased psychosocial problems in adolescence whilst those with combined day and night wetting (delayed and persistent classes) had more adverse outcomes. Previous studies have reported that children with combined wetting have more psychological problems than those with bedwetting alone [7, 10, 17, 18], but no studies have examined adolescent outcomes associated with combined wetting in childhood. Earlier studies reported associations between persistent bedwetting and adverse psychosocial outcomes in adolescence, but they did not distinguish between bedwetting alone versus bedwetting with daytime wetting [13, 14]. If children with combined wetting are more likely than those with either daytime wetting or bedwetting alone to experience a continuation or relapse in UI between ages 9–14, this might explain why combined wetting is associated with more psychosocial problems in adolescence. We did not have data on UI occurring between the ages of 9 and 14, so we were unable to ascertain whether UI persisted, re-emerged or resolved during that period. Only a small proportion (0.62%) of adolescents reported suffering from combined wetting and almost three quarters of these were wetting only occasionally (less than once a week). This might explain why we found less evidence for psychosocial problems in this group compared to those with either daytime wetting or bedwetting alone in adolescence.

The data on UI did not permit us to distinguish between primary (never reliably dry) and secondary enuresis (wetting again after a period of dryness lasting at least 6 months) due to the length of time (approximately a year) between the parental reports of UI. It has been argued, however, that the important factor relating bladder control to psychosocial problems is the age at which continence is attained, rather than whether the child experiences primary or secondary enuresis [14].

There was no information available on underlying anatomic or neurologic causes of incontinence in our sample, but the majority of cases of bedwetting and daytime wetting in children and adolescents are known to be functional [35]. We did not consider whether treatment for UI might have impacted on the findings. Parents were asked to report whether children had received treatment (bedwetting alarm or medication) for UI at ages 7 and 9 years, but only a small proportion of children (0.2–0.4%) had received treatment and there was no information on onset or duration of treatment.

### Interpretation of the findings

We find evidence that previous UI in childhood and current UI in adolescence are associated with increased levels of psychosocial problems in adolescence. An earlier prospective study reported higher levels of mental health disorders at ages 11 and 13 in children who had ceased bedwetting by age 11 compared with those who were dry by age 9 [13]. This suggests that children with delayed attainment of continence have lasting psychological problems into adolescence. It is possible that this finding is due to the comorbidity between delayed bladder control, general developmental delay [36] and behaviour problems (e.g. ADHD, conduct problems) [9]. However, the association persisted after we adjusted for early measures of developmental delay (at 18 months) and behaviour problems (at 3½ years) reported by parents. These assessments preceded the age at which incontinence is usually viewed as problematic by parents, therefore reducing the likelihood that parental reports were biassed by their child’s incontinence. However, many child psychiatric disorders become apparent at a later age, so the results do not rule out that later psychological problems in childhood could be predecessors of the problems in adolescence. Children with ADHD and other behavioural problems are more difficult to treat, which also contributes towards the risk of persistence of bedwetting and daytime incontinence [37].

The shame and stigma associated with incontinence may affect friendships and participation in social activities and this may increase the risk of psychosocial problems in adolescence even after continence problems have resolved. The presence of UI in adolescence is particularly distressing because this is the period during which identity and body image are formed, peer acceptance is valued and there is an increasing desire for autonomy from parents. Most young people with UI have a strong desire to conceal the problem from their peers and worry about being found out. Daytime wetting is often difficult to hide and leads to significant embarrassment and distress. This might explain why adolescents with daytime wetting had the highest level of psychosocial problems. Bedwetting is easier to conceal from peers, but was still associated with peer victimisation and poor self-image and females had increased levels of depressive symptoms. Fear of detection of bedwetting, parental stress and intolerance and worries about participating in school trips and sleepovers are all threaten the psychosocial development of young people [15]. There is evidence that adverse outcomes associated with persistent bedwetting continue through late adolescence and adulthood, with sufferers reporting higher rates of depression, lower self-esteem, lower educational attainment, absences from work and disruption of social activities [38].

### Clinical implications

Adolescence is a critical time of transition and psychosocial problems during this period have been linked to future adverse mental health outcomes. Poor self-image is related to low self-esteem [39] and depression [40] and these are well-established risk factors for subsequent mental health and social problems [41, 42]. Adolescents with depressive symptoms have an elevated risk of later depression and suicidal behaviours [43]. School-related problems in adolescence have adverse impacts on emotional health [30] and are related to self-harm [44]; peer victimisation in adolescence has an impact on depression in early adulthood [28]. Parents should be aware of possible long-term sequelae of childhood UI, even if the UI has resolved. Clinicians should enquire about psychosocial problems in teens presenting with UI and be aware that support from psychological services might be required to help prevent future mental health problems.

## Electronic supplementary material

Below is the link to the electronic supplementary material.

Supplementary material 1 (DOCX 49 kb)

Supplementary material 2 (DOCX 1634 kb)

Supplementary material 3 (DOCX 89 kb)

## References

[1] Jansson UB, Hanson M, Sillén U, Hellström AL (2005). Voiding pattern and acquisition of bladder control from birth to age 6 years—a longitudinal study. J Urol.

[2] Fergusson DM, Horwood LJ, Shannon FT (1986). Factors related to the age of attainment of nighttime bladder control: an 8-year longitudinal study. Pediatrics.

[3] Sureshkumar P, Jones M, Cumming R (2009). A population based study of 2856 school-age children with urinary incontinence. J Urol.

[4] Byrd RS, Weitzman M, Lanphear NE, Auinger P (1996). Bed-wetting in US children: epidemiology and related behaviour problems. Pediatrics.

[5] Butler RJ, Golding J, Northstone K (2005). Nocturnal enuresis at 7½ years old: prevalence and analysis of clinical signs. Br J Urol Int.

[6] Joinson C, Heron J, von Gontard A (2006). Psychological problems in children with daytime wetting. Pediatrics.

[7] Joinson C, Heron J, Emond A, Butler R (2007). Psychological problems in children with bedwetting and combined (day and night) wetting: a UK population-based study. J Pediatr Psychol.

[8] von Gontard A, Baeyens D, Van Hoecke E (2011). Psychological and psychiatric issues in urinary and fecal incontinence. J Urol.

[9] von Gontard A, Equit M (2015). Comorbidity of ADHD and incontinence in children—a review. Eur Child Adolesc Psychiatry.

[10] Thibodeau BA, Metcalfe P, Koop P, Moore K (2013). Urinary incontinence and quality of life in children. J Pediatr Urol.

[11] Theunis M, Van Hoecke E, Paesbrugge S, Hoebeke P, Vande Walle J (2002). Self-image and performance in children with nocturnal enuresis. Eur Urol.

[12] Joinson C, Sullivan S, von Gontard A, Heron J (2016). Early childhood psychological factors and risk for bedwetting at school age in a UK cohort. Eur Child Adolesc Psychiatry.

[13] Feehan M, McGee R, Stanton W, Silva PA (1990). A 6 year follow-up of childhood enuresis: prevalence in adolescence and consequences for mental health. J Paediatr Child Health.

[14] Fergusson DM, Horwood LJ (1994). Nocturnal enuresis and behavioral problems in adolescence: a 15-year longitudinal study. Pediatrics.

[15] Schulpen TW (1997). The burden of nocturnal enuresis. Acta Paediatr.

[16] Butler RJ, McKenna S (2002). Overcoming parental intolerance in childhood nocturnal enuresis: a survey of professional opinion. BJU Int.

[17] Berg I, Fielding D, Meadow R (1977). Psychiatric disturbance, urgency, and bacteriuria in children with day and night wetting. Arch Dis Child.

[18] Van Hoecke E, De Fruyt F, De Clercq B (2006). Internalizing and externalizing problem behavior in children with nocturnal and diurnal enuresis: a five-factor model perspective. J Pediatr Psychol.

[19] Croudace TJ, Jarvelin MR, Wadsworth ME (2003). Developmental typology of trajectories to nighttime bladder control: epidemiologic application of longitudinal latent class analysis. Am J Epidemiol.

[20] Joinson C, Heron J, Butler R (2009). Development of nighttime bladder control from 4 to 9 years: association with dimensions of parent rated child maturational level, child temperament and maternal psychopathology. LLCS.

[21] Sullivan S, Joinson C, Heron J (2015). Factors predicting atypical development of nighttime bladder control: a prospective cohort study. J Dev Behav Pediatr.

[22] Heron J, Joinson C, Croudace T, von Gontard A (2008). Trajectories of daytime wetting and soiling in a United kingdom 4- to 9-year-old population birth cohort study. J Urol.

[23] Joinson C, Grzeda M, von Gontard A, Wright A, Heron J (2016). The association between trajectories of bedwetting and daytime wetting in childhood and incontinence and lower urinary tract symptoms in adolescence. Arch Dis Child.

[24] Boyd A, Golding J, Macleod JAA (2013). Cohort profile: the ‘Children of the 90s’—the index offspring of the avon longitudinal study of parents and children. Int J Epidemiol.

[25] Angold A, Costello EJ, Messer SC (1995). The development of a short questionnaire for use in epidemiological studies of depression in children and adolescents. Int J Methods Psychiatr Res.

[26] Thapar A, Guffin P (1998). Validity of the shortened Mood and Feelings Questionnaire in a community sample of children and adolescents: a preliminary research note. Psychiatry Res.

[27] Turner N, Joinson C, Peters TJ, Wiles N, Lewis G (2014). Validity of the short mood and feelings questionnaire in late adolescence. Psychol Assess.

[28] Bowes L, Joinson C, Wolke D, Lewis G (2015). Peer victimisation during adolescence and its impact on depression in early adulthood: prospective cohort study in the United Kingdom. BMJ.

[29] Butler RJ (2001) Self-image profiles: manual. Pearson Assessment, Halley court, Jordan Hill, Oxford OX2 & EJ

[30] Kidger J, Heron J, Leon DA (2015). Self-reported school experience as a predictor of self-harm during adolescence: a prospective cohort study in the South West of England (ALSPAC). J Affect Disord.

[31] Frankenburg WK, Dodds J, Archer P, Shapiro H, Bresnick B (1992). The Denver II: a major revision and restandardization of the Denver Developmental Screening Test. Pediatrics.

[32] Elander J, Rutter M (1996). Use and development of the Rutter parents’ and teachers’ scales. Int J Methods Psychiatr Res.

[33] Asparouhov T, Muthen BO (2013) Auxiliary Variables in Mixture Modeling: 3-Step Approaches Using Mplus. Mplus Web Notes: No 15. https://www.statmodel.com/download/webnotes/webnote15.pdf. Accessed 11th July 2016

[34] Bakk Z, Tekle FB, Vermunt JK (2014). Estimations the association between latent class membership and external variables using bias-adjusted three-step approaches. Sociol Methodol.

[35] von Gontard A, Nevéus T (2006). Management of disorders of bladder and bowel control in childhood.

[36] Jarvelin MR (1989). Developmental history and neurological findings in enuretic children. Dev Med Child Neurol.

[37] Baeyens D, Roeyers H, Demeyere I (2005). Attention-deficit/hyperactivity disorder (ADHD) as a risk factor for persistent nocturnal enuresis in children: a 2-year follow-up study. Acta Paediatr.

[38] Yeung CK, Sihoe JD, Sit FK, Bower W, Sreedhar B, Lau J (2004). Characteristics of primary nocturnal enuresis in adults: an epidemiological study. BJU Int.

[39] Bailey JA (2003). The foundation of self-esteem. J Natl Med Assoc.

[40] Fine S, Haley G, Gilbert M, Forth A (1993). Self-image as a predictor of outcome in adolescent major depressive disorder. J Child Psychol Psychiatry.

[41] Trzesniewski KH, Donnellan MB, Moffitt TE (2006). Low self-esteem during adolescence predicts poor health, criminal behavior, and limited economic prospects during adulthood. Dev Psychol.

[42] Keenan-Miller D, Hammen CL, Brennan PA (2007). Health outcomes related to early adolescent depression. J Adolesc Health.

[43] Fergusson DM, Horwood LJ, Ridder EM, Beautrais AL (2005). Subthreshold depression in adolescence and mental health outcomes in adulthood. Arch Gen Psychiatry.

[44] Kidger J, Heron J, Lewis G, Evans J, Gunnell D (2012). Adolescent self-harm and suicidal thoughts in the ALSPAC cohort: a self-report survey in England. BMC Psychiatry.

