# Supplementary material for: Effects of urinary incontinence on psychosocial outcomes in adolescence
Source: Eur Child Adolesc Psychiatry. 2016 Dec 10;26(6):649–58. doi: 10.1007/s00787-016-0928-0 (PMC5446552; doi:10.1007/s00787-016-0928-0)
Supplement: Supplementary file 1 — Supplementary material 1 (DOCX 49 kb) [file 787_2016_928_MOESM1_ESM.docx]

**Online Resource Part 1**

Article title: Effects of urinary incontinence on psychosocial outcomes in adolescence

# Journal name: European Child & Adolescent Psychiatry

Author names: Mariusz T Grzeda MSci^1^, Jon Heron PhD^1^, Alexander von Gontard MD PhD ^2^, Carol Joinson PhD^1^

Corresponding author: Carol Joinson, School of Social and Community Medicine, University of Bristol, Oakfield House, Oakfield Grove, Clifton, Bristol BS8 2BN, UK. Email: [Carol.Joinson@bristol.ac.uk](mailto:Carol.Joinson@bristol.ac.uk)

**Statistical analysis used to estimate the latent classes of daytime wetting and bedwetting**

This supplementary material presents the methods we employed to extract the latent classes of daytime wetting and bedwetting at 4 – 9 years using longitudinal latent class analysis (LLCA). LLCA is an empirical method, which uses a categorical latent variable to explain the associations observed between a set of binary or categorical repeated measures. The assumption is that patterns in the observed data are due to the presence of unobserved latent classes. The goal is to determine the number of these latent classes and estimate both the relative size of the classes (the class-distribution) and the developmental pattern exhibited by members of each latent class (the trajectory).

Daytime wetting and bedwetting data were obtained from parental reports when the study children were 4^1^/_2_, 5^1^/_2_, 6^1^/_2_, 7^1^/_2_, and 9^1^/_2_ years. Parents were asked “How often usually does your child wet the bed?” and were given the options “never”; “less than once a week”; “about once a week”; “2 to 5 times a week”; “nearly every night”; “more than once a night.” For daytime wetting, we dichotomised the data to produce a set of binary variables indicating those with daytime wetting (coded 1) or without daytime wetting (coded 0) at each time point. Due to the greater number of children wetting the bed at each age, we derived a three-category ordinal measure for the bedwetting data: no bedwetting (never: coded 0), bedwetting *‘*less than once a week’ and ‘about once a week’ (infrequent: coded 1) and bedwetting at least twice a week (i.e. any of the three most frequent response options: coded 2).

To illustrate this approach, Child A might have responses “10000” for the questions on daytime wetting at 4½, 5½, 6½, 7½, and 9½ years respectively, whereas child B might have responses “11111”. Such variability in these patterns of responses is accounted for by a latent factor that groups together children exhibiting similar patterns of development. The result is a number of different latent classes (trajectories). Each class can be seen to exhibit its own trajectory through time by plotting age against the class-specific prevalence of daytime wetting at each time point. Since some children are more easily assigned to a particular class than others, the output from a latent class model is a set of posterior probabilities describing the likelihood of a child being a member of each class. For instance, child A would be likely to have a high probability of being assigned to a group characterized by normal or slightly delayed development, whilst child B would have a higher probability of being a member of a group characterized by persistent daytime wetting.

The LLCA model assumes that observed heterogeneity in responses is due to a latent (unobserved) grouping in the population. Starting with a single class, additional classes are added until the various assessments of model fit reach an acceptable level. Similar to factor analysis, this latent categorical variable should explain the associations within the set of repeated measures such that within each latent class, respondents form a single homogeneous group, and furthermore the repeated measurements are independent of each other, i.e. there is local (conditional) independence.

Previously, we applied longitudinal latent class analysis (LLCA) separately to daytime wetting^1^ and bedwetting^2^ data collected from children in the ALSPAC cohort at 4 – 9 years. The classes we extracted are described below:

*Daytime wetting classes*

1. Normative development (86.2% of the sample): attainment of daytime bladder control at age 4 – 5 and very low probability of daytime wetting at any age;
2. Delayed (6.9%): delayed attainment of daytime bladder control characterised by steadily decreasing prevalence of daytime wetting from 80% at age 4.5 years to 40% at 6.5 years, to less than 10% by 9.5 years;
3. Relapsing (3.2%): initial normal development of daytime bladder control, but with an increased probability of daytime wetting at age 6 – 7, followed by a decreased probability up to age 9;
4. Persistent (3.7%): relatively high probability of daytime wetting from 4 – 9 years.

*Bedwetting classes*

1. Normative: (71.5% of the sample): low probability of bedwetting at any time point;
2. Infrequent delayed (14.3%): delayed attainment of nighttime bladder control and decreasing probability of infrequent (<twice a week) bedwetting from 4 – 9 years;
3. Infrequent persistent (8.6%): relatively high probability of infrequent bedwetting;
4. Frequent delayed (2.4%): high probability of frequent (>= twice a week) bedwetting at age 4 years, which decreased and became more infrequent at 6 – 9 years;
5. Frequent persistent (3.2%): relatively high probability of bedwetting at least twice a week from 4 – 9 years.

*Parallel latent class model of daytime wetting and bedwetting*

Deriving *separate* latent class models for daytime wetting and bedwetting ignores the comorbidity between these two continence problems. We derived a “parallel LLCA” in order to describe the repeated bivariate data of daytime wetting and bedwetting. The procedure that we followed to apply the parallel LLCA model to the daytime wetting and bedwetting data consisted of the following stages. First, we started our analyses by deriving separate LLCA models for daytime and nighttime wetting indicators (see above). We then derived the parallel LLCA model including daytime wetting and bedwetting simultaneously.

To determine the optimal number of latent classes of daytime wetting and bedwetting, we estimated parallel LLCA models including all possible combinations of number of classes and analysed each solution with respect to three statistical criteria: Bayesian information criterion (BIC) ^3^; entropy^4^ and the analysis of the standardized residuals and overall bivariate Pearson chi square statistics associated with them. It is rare that all indicators of model fit point to the same solution. The accepted approach is to evaluate the statistical evidence alongside face validity, resemblance to other results in the literature, and pragmatic issues such as class size.

The final parallel LLCA solution that we selected had 4 classes for daytime wetting and 5 classes for bedwetting. This model had a good fit and adequately explained the longitudinal heterogeneity in development of both daytime and nighttime bladder control. The four-by-five class solution represented 20 separate subgroups corresponding to each combination of daytime wetting and bedwetting. It is not practical to examine the risk of each adolescent outcome within each of these groups. Consequently we collapsed these groups into five distinct, clinically relevant classes (see table S1).

**References**

1. Heron J, Joinson C, Croudace T, von Gontard A. Trajectories of daytime wetting and soiling in a United Kingdom 4 to 9-year-old population birth cohort study. *Journal of Urology*. 2008;179:1970-1975.

2. Sullivan S, Joinson C, Heron J. Factors predicting atypical development of nighttime bladder control: a prospective cohort study. *J Dev Behav Pediatr*. 2015;36(9):724-33.

3. Schwarz G. Estimating the dimension of a model. *Ann Stat*. 1978;6:461–464.

4. McCutcheon AL. *Latent Class Analysis* (Sage University Paper series on Quantitative Applications in Social Sciences, No. 07-064). Newbury Park, CA: Sage; 1987.

**Table S1. Description and prevalence of the parallel latent classes of daytime wetting and bedwetting (n = 8,751)**

| Class name | Description | Number in each class (%) | % males, % females |
| --- | --- | --- | --- |
| Normative development of bladder control | Comprises normative classes for both daytime wetting and bedwetting. | 5513 (63.0%) | 47.5%, 52.5% |
| Delayed attainment of bladder control | Comprises infrequent and frequent delayed bedwetting classes and the non-normative daytime wetting classes. | 752 (8.6%) | 52.9%, 47.1% |
| Bedwetting alone (no daytime wetting) | Comprises all non-normative bedwetting classes and the normative daytime wetting class. | 1365 (15.6%) | 68.4%, 31.6% |
| Daytime wetting alone (no bedwetting) | Comprises all non-normative daytime wetting classes and the normative bedwetting class. | 508 (5.8%) | 33.5%, 66.5% |
| Persistent wetting (persistent bedwetting with daytime wetting) | Comprises the infrequent persistent and frequent persistent bedwetting classes and the non-normative daytime wetting classes. | 613 (7.0%) | 63.0%, 37.0% |
