# Supplementary material for: Effects of urinary incontinence on psychosocial outcomes in adolescence
Source: Eur Child Adolesc Psychiatry. 2016 Dec 10;26(6):649–58. doi: 10.1007/s00787-016-0928-0 (PMC5446552; doi:10.1007/s00787-016-0928-0)
Supplement: Supplementary file 2 — Supplementary material 2 (DOCX 1634 kb) [file 787_2016_928_MOESM2_ESM.docx]

**Online Resource Part 2**

*Article title:* Effects of urinary incontinence on psychosocial outcomes in adolescence

# *Journal name:* European Child & Adolescent Psychiatry

*Author names:* Mariusz T Grzeda MSci^1^, Jon Heron PhD^1^, Alexander von Gontard MD PhD ^2^, Carol Joinson PhD^1^

*Corresponding author:* Carol Joinson, School of Social and Community Medicine, University of Bristol, Oakfield House, Oakfield Grove, Clifton, Bristol BS8 2BN, UK. Email: [Carol.Joinson@bristol.ac.uk](mailto:Carol.Joinson@bristol.ac.uk)

# **Details on the Rasch estimation**

In this supplement we provide a brief theoretical description of the Rasch approach and its basic extension that we applied in this paper. This is followed by the specification of the strategy we adopted here to validate the scales used as outcomes in the model including the parallel LLCA. Finally, we show the performance and scaling properties of the scales that we developed.

## *Why Rasch modeling?*

Since variables gathered by ALSPAC questionnaires were ordinal there arose a problem of obtaining a valid transformation to turn them into a single continuous measure representing a theoretical concept. The simplest strategy of adding up all categories across all items is inappropriate for at least two reasons:

1. Such summary scores represent merely the counts of categories and are indicators only of the order among respondents and cannot necessarily be treated as interval variables;
2. There is also a problem with the underlying premise of that operation, assuming that all variables included represent one dimension (the same concept). In fact this assumption might be problematic and rather should be carefully checked against data rather than simply taken-for-granted (Bond & Fox, 2007; Wright & Stone, 1979).

There are number of solutions proposed in the literature to the problem of valid transition from ordinal variables to interval scales. In this paper we used the solution based on the idea proposed by Georg Rasch. Although this original model concerned only dichotomous variables with ordered categories, it was extended very soon to polytomous cases. This extension was devised by David Andrich and further by Geoff Masters (Bond & Fox, 2007; Wright & Masters, 1982; Wright & Stone, 1979).

*Rasch dichotomous model*

Briefly, the Rasch dichotomous model assumes that categories on ordinal variables are observable manifestations of a latent continuous variable. The model relies on the assumption that ‘0’ indicating ‘less’ of the trait represented by latent continuum and ‘1’ indicating ‘more’. It is assumed that they are separated by a threshold located on the continuum. It is also assumed that the latent variable is common to both cases measured (respondents) and for items (indicators of theoretical concept). In other words, both respondents and items can be located on the same continuum.

The level of the theoretical concept is denoted by$\beta$, and the level of item difficulty by $\delta$. Next, it is also assumed that there exists a probabilistic function that relates the level of latent trait possessed by each individual ($\beta$) with the level the difficulty of each item ($\delta$). This function links the probability of a particular answer on an item with the difference between $\beta$ and $\delta$.

The Rasch model takes the form of a logistic function [1]:

$P(X_{ij}=x|\beta_{i},\delta_{j})=\frac{\mathrm{e}^{(x(\beta_{i}-\delta_{j}))}}{1+\mathrm{e}^{^{(\beta_{i}-\delta_{j})}}}$ [1]

where: X is the observable indicator of the latent measure.[[1]](#footnote-1)

Since Rasch’s seminal work, his dichotomous model has been expanded quickly and in a few decades revolutionized measurement of theoretical constructs in the social behavioral and medical sciences (Merbitz et al., 1989; Penta, Tesio, Arnould, Zancan, & Thonnard, 2001).

*Further extensions to polytomous case*

The model created by Rasch was generalized for items with more than two categories (Wright & Masters, 1982). These generalizations are called Rating Scale Model (RSM) and Partial Credit Model (PCM), respectively. The first assumes that all items included in the scale have the same structure while the second allows for differences across items. Both models work in a very similar way to the dichotomous Rasch model. There are only some minor differences: (1) in this case more than two categories are indicating successively ordered levels of a theoretical concept. So the answers for items are coded from ‘0’ through ‘1’ up to ‘m’ to represent increasing levels of theoretical concept being measured. Consequently, in this case it is also assumed that categories are separated by k thresholds (k=m-1) located in certain points of the latent continuum. (2) The function by which RSM and PCM are linking the probability of observing response *x* on the item X, given the respondent specific ability level of $\beta_{i}$ and the item’s difficulty $\delta_{j}$, is given by the following formulae:

$P(X_{ij}=x|\beta_{i},\delta_{j})=\frac{\mathrm{e}^{\sum_{k=0}^{x} (\beta_{i}-\delta_{j}-\tau_{k})}}{\sum_{x=0}^{m} e^{\sum_{k=0}^{x} (\beta_{i}-\delta_{j}-\tau_{k})}}$ [2]

for: $\begin{matrix} x=0,1,...,m \\ k=1,..,m \\ \tau_{k=0}\equiv0 \end{matrix}$

In the model above, a category specific parameter is introduced, $\tau_{k}$ , that is difficulty threshold parameter for category k. In RSM $\tau_{k}$are the same for all items, while in PCM $\tau_{k}$ parameters are free to vary across items (and therefore in PCM thresholds $\tau_{k}$ are represented with the additional index *j* ). For more thorough explanation of these models we refer to (Wright & Masters, 1982).

## *Data analysis*

Since the items conceived to represent psychological scales included in this article had more than two categories we needed to choose between Partial Credit Model (PCM) and Rating Scale Model (RSM). We decided to adopt the PCM here. The reason for this was that there were no a priori arguments suggesting that the interval separating responses was the same across all the items. This more flexible approach allowed us for more thorough screening of items. The sample size was sufficiently large to support this decision.

## *Fitting the Rasch model*

The elements of our Rasch analysis of each psychological concept included checking whether the fundamental assumptions of the theoretical model were fulfilled in the data. Specifically we checked: category thresholds ordering (ensuring that increase in categories with corresponding thresholds reflects an increase in the underlying trait); examination of fit statistics (Infit/Outfit Mean Square indexes of which values between 0.7 and 1.3 were considered as indicating acceptable item fit (Wright & Linacre, 1994) (Smith, Rush et. al 2008));  point-measure correlations *(*correlations between item scores and values on Rasch continuum of at least 0.40 were considered as satisfactorily fulfillment of the assumptions of the model; this criterion was applied with caution especially to the items of extreme difficulty as it is known that they have a tendency to have lower correlations with Rasch continuum); item descriptive statistics (comparisons of means computed on the continuum across all categories for each item; averages for each successive categories should be increasing (Linacre & Box, 2008) (Schulz and Fraillon, 2008); check for different item functioning DIF (the hierarchies of item difficulties were compared across groups of respondents; we applied ETS classification accepting low/moderate DIFs <|0.63|); uni-dimensionality (principal component analysis (PCA) of residuals were performed as the procedure for checking of the assumption of uni-dimensionality. We applied the criterion stating that the highest eigenvalue obtained from this analysis should not exceed 2.0; eigenvalues above this threshold are suggesting the existence of more than one dimension. Then closer inspection of the pattern of item loadings obtained from PCA is recommended to look for alternative dimensions underlying the set of items (Linacre, 2011).; reliability indices (person reliability index and separation index. Reliability of .70 indicate a satisfactory scale reliability and values over .80 an good reliability and over .90 excellent).

All items that showing misfitting to the assumptions of the Rasch model were excluded iteratively from the pool of items considered as indicators of each theoretical concept. In each itera­tion the assumptions were re-checked for all items. At every step we were removing only one, most problematic item.

## *Scales*

By application of the above procedure we have developed 4 scales. These are: Self-Image (SIS), Perception of School (POS), Perception of Teachers (POT) and Social Relations at School (SRaS). In the remaining cases the psychometric properties of scales obtained were not sufficiently good (activity in leisure time and satisfaction with friends), so we made the decision not to include them in further analyses. Below, we are presenting final item sets for the scale that achieved desirable level of consistency with expectations of theoretical model applied here (PCM).

## **Self Image Scale (SIS)**

The first scale we developed here concerns self-image of the teenager. This measure was obtained from an initial pool of 25 questions. Items within this scale explore several aspects of auto perception of the teenager. Respondents were asked how frequently they feel that they are certain types of person or have certain traits. Through the Rasch analysis we reduced the number of items to 7. As it is specified in Tables A1 and A2 the scale has satisfactory psychometric properties. As Figure A1 shows the category thresholds were ordered as expected; all misfitting indices were in the assumed bands of 0.7-1.3 and all point-measure correlations were positive and relatively high (>.40). Test of uni-dimensionality (PCA on residuals from the model) confirmed that the model is unidimensional (highest eigenvalue <=2.0). No problems with DIF were presented. The scale turned out to have a good level of reliability .74. Figure A2 shows map of the results obtained from the final set of 7 items in a form of person-item Wright map. The final items are well targeted for that population. The spread of threshold categories was approximately uniform and the range of them is near 6 logits, which indicates sufficiently wide scale. The ceiling and floor effects were marginal.

**Table A1.** Final item set representing self-image scale (SIS). The table includes item parameters (difficulty, Rasch-Andrich cumulative thresholds) obtained from the final model as well as basic indicators of item fit and discrimination (Infit, Outfit, point-measure correlation). The summary of indexes used to assess the reliability of final SIS items is shown below the table.

| Item | Rasch measure  – item difficulty (SE) | Infit MnSQ | Outfit MnSQ | Point measure  correlation | Rasch-Andrich thresholds  (50% cumulative thresholds) | | | |
| --- | --- | --- | --- | --- | --- | --- | --- | --- |
| Frequency of feeling… |  |  |  |  | 1 | 2 | 3 | 4 |
| confident | .43 (.02) | .79 | .79 | .68 | -2.08 | -.37 | 1.29 | 2.88 |
| sporty | .20 (.01) | 1.02 | 1.06 | .61 | -.87 | -.55 | .53 | 1.69 |
| intelligent | .49 (.02) | 1.18 | 1.19 | .44 | -2.25 | .05 | 1.75 | 2.40 |
| good looking | -.44 (.02) | .96 | .95 | .64 | -2.35 | -1.34 | .67 | 1.25 |
| shy (rev.) | .13 (.02) | .97 | .97 | .58 | -2.79 | -.73 | 1.50 | 2.54 |
| different from others (rev.) | -.45 (.02) | 1.09 | 1.10 | .55 | -2.83 | -1.20 | .94 | 1.30 |
| worries a lot (rev.) | -.36 (.02) | .98 | .98 | .61 | -3.00 | -.52 | .68 | 1.42 |

Person reliability index=.74 ; separation index=1.67 raw score-Rasch measure correlation coefficient=.96

*rev*.: categories of that items were reversed (see section on recoding)

**Figure A1.** Category probabilities curves for SIS final items


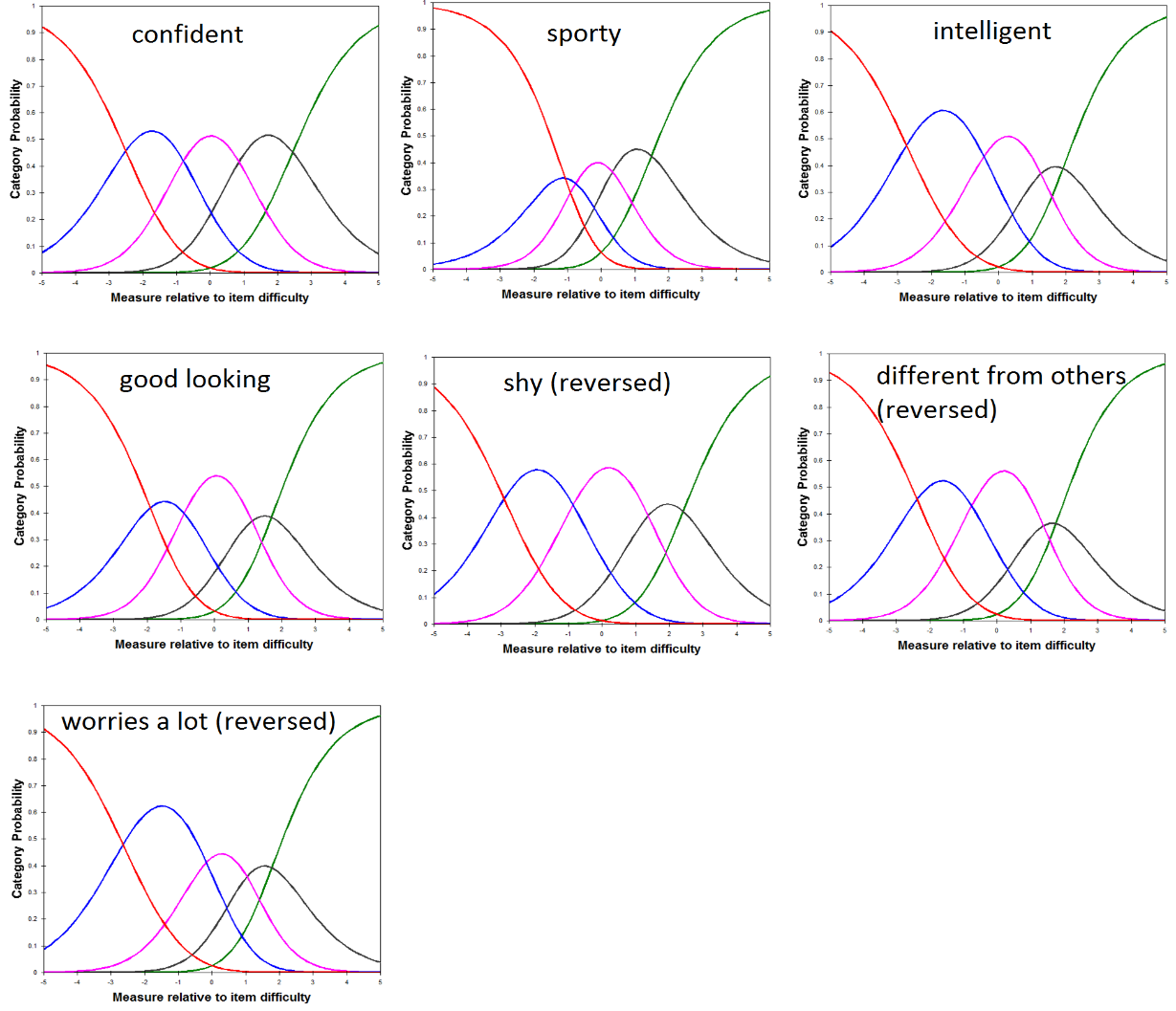


**Figure A2 (overleaf).** Person-item map (also known as a *Wright map*) of the Self-Image Scale (SI). Vertical line in the middle of the figure depicts graphically the SIS variable of which units are logits. Since categories of each item were reversed before being scaled the higher values of this variable represent worse (more negative) self-image while the lower better (more positive) self-image. On the left side of this line individual patients are presented according to their level of SIS (each # represents 46 respondents while each ‘.’ represents 1 up to 45 cases). On the right side of the vertical line the Rasch-Andrich thresholds are shown. They represent the points on the Rasch continuum at which two adjacent response categories of particular item have the same probability of being answered. Every threshold on the map is represented by the item code (see the note below the figure) followed by a dot, and the particular threshold number informing which transition point it refers to. (Thus, in example point ccp130_r5.2 on the map represents the level of SIS at which categories coded as 1 and 2 of that item are equally probable; point ccp130_r5.3 the point of transition between categories 2 and 3 and so on). The *M* points on the map are corresponding to the means (mean level of SIS on the left side of the vertical line and mean item difficulty on the right – by convention set at 0). The remaining letter points ‘*S*’ and ‘*T*’ corresponds to 1 and 2 standard deviation respectively from their respective means.


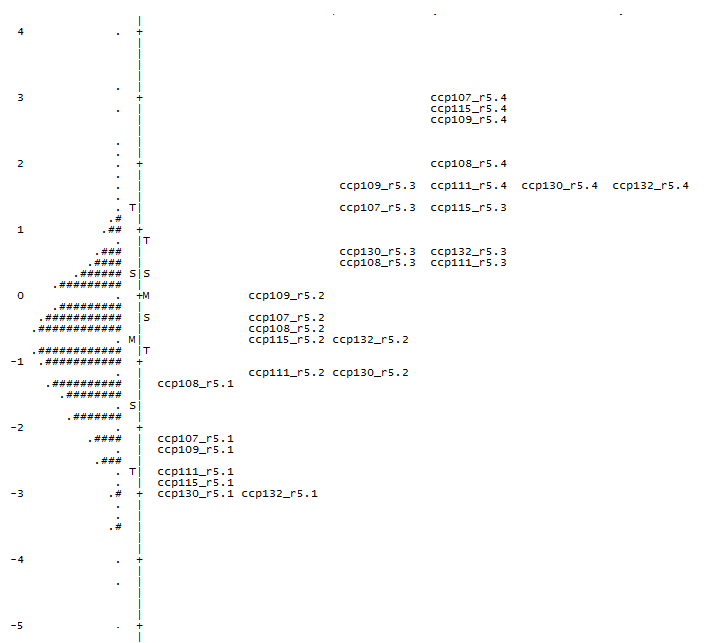


*The following abbreviations for items are adapted above:*

*ccp107_r5 (Confident);*

*ccp108_r5 (Sporty);*

*ccp109_r5 (Intelligent);*

*ccp111_r5 (Good looking),*

*ccp115_r5 (Shy);*

*ccp130_r5 (Different from others);*

*ccp132_r5 (Worries a lot).*

**Table A2.** Rasch measurement indexes obtained from the final solutions of scales SI=self-image; PoS=perception of School; PRaS=Peer relations at School; PoT=Perception of teachers

| Model summary statistics | SIS | PoSS | SRaS | PoT |
| --- | --- | --- | --- | --- |
| **Variance explained** |  |  |  |  |
| Total variance explained by the measures | 39.3% | 65.0% | 56.1% | 44.5% |
| Variance explained by respondents | 17.3% | 49.8% | 54.5% | 29.6% |
| Variance explained by items | 21.9% | 15.2% | 1.6% | 14.9% |
|  |  |  |  |  |
| **Residual variance** |  |  |  |  |
| Variance unexplained | 60.7% | 35.0% | 35.0% | 55.5% |
|  |  |  |  |  |
| **Principal Component Analysis of residuals** |  |  |  |  |
| 1^st^ contrast eigenvalue | 1.6 | 1.6 | 1.4 | 2.0 |
| 2^nd^ contrast eigenvalue | 1.5 | 1.4 | 1.3 | 1.5 |
| 3^rd^ contrast eigenvalue | 1.2 | 1.2 | 1.2 | 1.4 |
| 4^th^ contrast eigenvalue | 1.1 | 1.0 | 1.1 | 1.2 |
| 5^th^ contrast eigenvalue | .9 | .9 | 1.0 | 1.1 |

## **Perception of School (PoS) and Social Relations at School (SRaS)**

The next scale that was successfully developed here was the scale pertaining to the perception of school (POS) by 14 year old teenagers. Initially we tried to develop this scale relying on the pool of 20 items asking about certain aspects of school life. This initial set of items included statements specified in Table A3. Possible answers to all of them were: *strongly agree, agree, disagree, strongly disagree*, coded by 1,2,3,4 respectively. These category codes were first recoded to the start from 0 and end at 3. Next, codes of all items referring to negative feelings (*lonely, worried, upset*) were reversed to make their order consistent with the remaining (positive) items. Scale was deemed that way that higher values should indicate worse perception of school. After couple of iterations reducing the initial pool of items (by removing those misfitting) it became clear that two dimensions are underlying the poll of items. We decided to develop two scales for them.

First dimension distinguished was perception of school (PoS) that tapped the overall image of the school as a institution perceived by the teenagers while another was covering something that we finally named quality of social relations at school (PRaS).

We based our decision about distinguishing these two dimensions on the high value eigenvalue (2.7) clearly suggesting more than one dimension obtained from PCA conducted on model residuals. The inspection of PCA factor loadings revealed this clear and interpretable pattern of two separate dimensions (not shown). When we decided to split these items it turned out that both subsets analyzed separately have very good psychometric properties.

Items that were kept as representing the PoS and SRaS as separate scales did not revealed any problems with the threshold ordering (see Figure A3 & A4). The category averages were also not reversed (not shown). All final items for these two scales have misfitting indices within acceptable range. SRaS slightly better than PoS. One item PoS (get excited about the work they do) had Outfit index at the level of 1.29 which was in fact borderline case. In case of SRaS all items had misfitting indexes closer to 1. The point measure correlations computed for these items also were very high (all above .70 for PoS and only slightly lower for PRaS) (Tables A4 & A5). No problematic DIF has occurred. The reliability indexes were satisfactory good for those two scales: .86 .78 for PoS and PRaS respectively. Both scales are clearly unidimensional having highest eigenvalues far below the accepted threshold. The potential disadvantage of both scales is the fact that the threshold categories are not distributed uniformly and both scales have a gap (PoS rather small but PRaS larger) in the middle of the Rasch continuum. This might cause problems with discriminating pupils at this levels of the trait. Anyway this fact seems to not be degrading the overall good psychometric properties of these scales.

**Table A3.** Initial item set exploring questions about the school.

| School items |  |  |  |  |  | |
| --- | --- | --- | --- | --- | --- | --- |
| … they really like to go each day |  |  |  |  |  |
| …they learn to get along with other people |  |  |  |  |  |
| …other pupils accept them |  |  |  |  |  |
| … they like to be |  |  |  |  |  |
| … they like to do extra work |  |  |  |  |  |
| …they feel happy |  |  |  |  |  |
| …they feel lonely (negative) |  |  |  |  |  |
| …they feel proud to be a pupil |  |  |  |  |  |
| …they feel worried (negative) |  |  |  |  |  |
| …where people trust each other |  |  |  |  |  |
| … they have a lot of fun |  |  |  |  |  |
| … they enjoy what they do in class |  |  |  |  |  |
| …they can learn what they need to know |  |  |  |  |  |
| … they get excited about the work they do |  |  |  |  |  |
| …they get upset (negative) |  |  |  |  |  |
| …they know people who think a lot of me |  |  |  |  |  |
| …they get on well with other pupils |  |  |  |  |  |
| ….people can depend on them |  |  |  |  |  |
| …other pupils are very friendly |  |  |  |  |  |
| …they feel restless |  |  |  |  |  |

**Table A4.** Final item set representing Perception of School scale (PoS). The table includes item parameters and basic indicators of item fit and discrimination (Infit, Outfit, point-measure correlation) obtained from the final model. Reliability indexes of scale are shown below the table.

| Item | Rasch measure  – item difficulty (SE) | Infit MnSQ | Outfit MnSQ | Point measure  correlation | Rasch-Andrich thresholds  (50% cumulative thresholds) | | |
| --- | --- | --- | --- | --- | --- | --- | --- |
| **School is a place where they…** |  |  |  |  | 1 | 2 | 3 |
| … really like to go each day | -.55 (.03) | .80 | .75 | .82 | -5.89 | .25 | 4.00 |
| … like to be | .05 (.03) | .80 | .73 | .82 | -4.80 | 1.08 | 3.87 |
| … feel happy | 1.36 (.04) | .89 | .85 | .75 | -3.97 | 2.84 | 5.21 |
| … feel proud to be a pupil | .07 (.04) | 1.14 | 1.10 | .75 | -5.10 | .86 | 4.45 |
| … have a lot of fun | 1.34 (.03) | 1.08 | 1.07 | .73 | -3.31 | 2.13 | 5.20 |
| … enjoy what they do | .29 (.04) | .92 | .82 | .77 | -5.75 | 1.27 | 5.35 |
| … get excited about the work they do | -2.57 (.03) | 1.21 | 1.29 | .72 | -8.02 | -2.53 | 2.85 |

Person reliability index=.86 ; separation index=2.51 raw score-Rasch measure correlation coefficient=.89

**Figure A3.** Category probabilities curves for PoS items obtained from the final solution


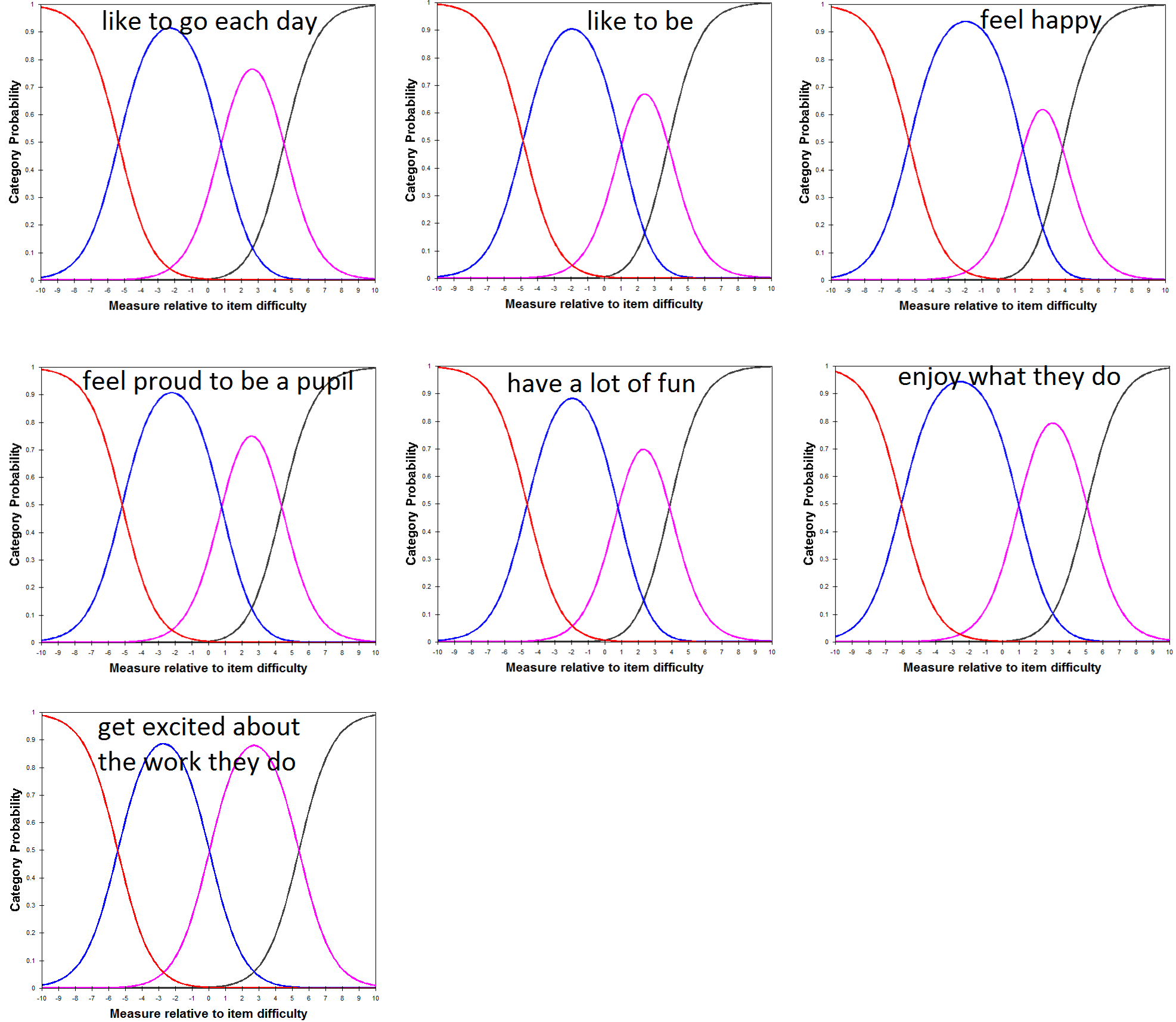


**Figure A4.** Person-item map of the Perception of School Scale (PoS). For the detailed interpretation of this map see the description provided in the previous section for the SI

*
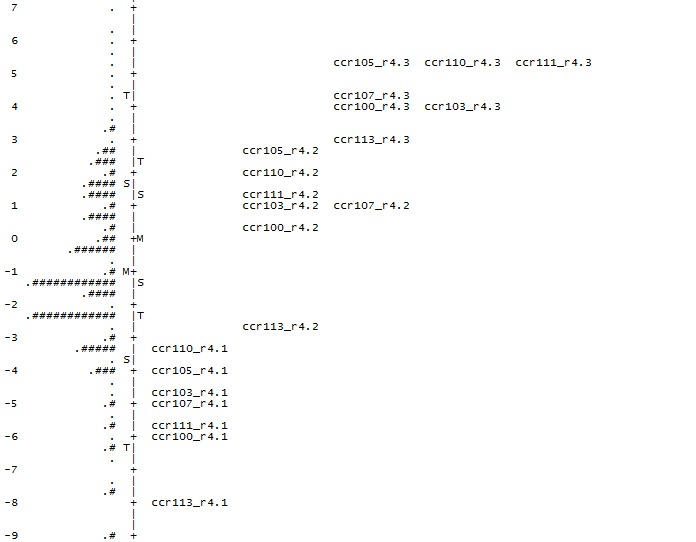
*

*The following abbreviations for items are adapted:*

*ccr100_r4 (school as a place they like to go each day);*

*ccr103_r4 (… like to be );*

*ccr105_r4 (… feel happy);*

*ccr107_r4 (…feel proud to be a pupil),*

*ccr110_r4 (…have a lot of fun);*

*ccr111_r4 (…enjoy what they do);*

*ccr113_r4 (…get excited about the work they do).*

**Table A5.** Final item set representing Peer relations at School Scale (PRaS). The table includes item parameters and basic indicators of item fit and discrimination (Infit, Outfit, point-measure correlation) obtained from the final model. Reliability indexes of scale are shown below the table.

| Items  **Peer Relations at School** | Rasch measure  – item difficulty (SE) | | Infit MnSQ | Outfit MnSQ | Point measure  correlation | Rasch-Andrich thresholds  (50% cumulative thresholds) | | |
| --- | --- | --- | --- | --- | --- | --- | --- | --- |
| **School is a place where they…** |  | |  |  |  | 1 | 2 | 3 |
| … learn to get along with other people | | .70 | 1.18 | 1.08 | .67 | -5.29 | 2.38 | 5.01 |
| … other pupils accept them | | -.37 | .91 | .82 | .78 | -5.16 | 1.06 | 2.99 |
| … know people who think a lot of me | | -1.28 | 1.09 | 1.13 | .73 | -7.00 | -.16 | 3.31 |
| … place where they get on well with other pupils | | .86 | .77 | .65 | .79 | -4.09 | 2.62 | 4.05 |
| … people can depend on them | | .51 | 1.08 | 1.02 | .68 | -5.51 | 2.32 | 4.71 |
| … other pupils are very friendly | | -.42 | .86 | .77 | .79 | -5.40 | .81 | 3.33 |

Person reliability index=.78 ; separation index=1.86 raw score-Rasch measure correlation coefficient=.90

**Figure A4.** Category probabilities curves for PRaS items obtained from the final solution

*
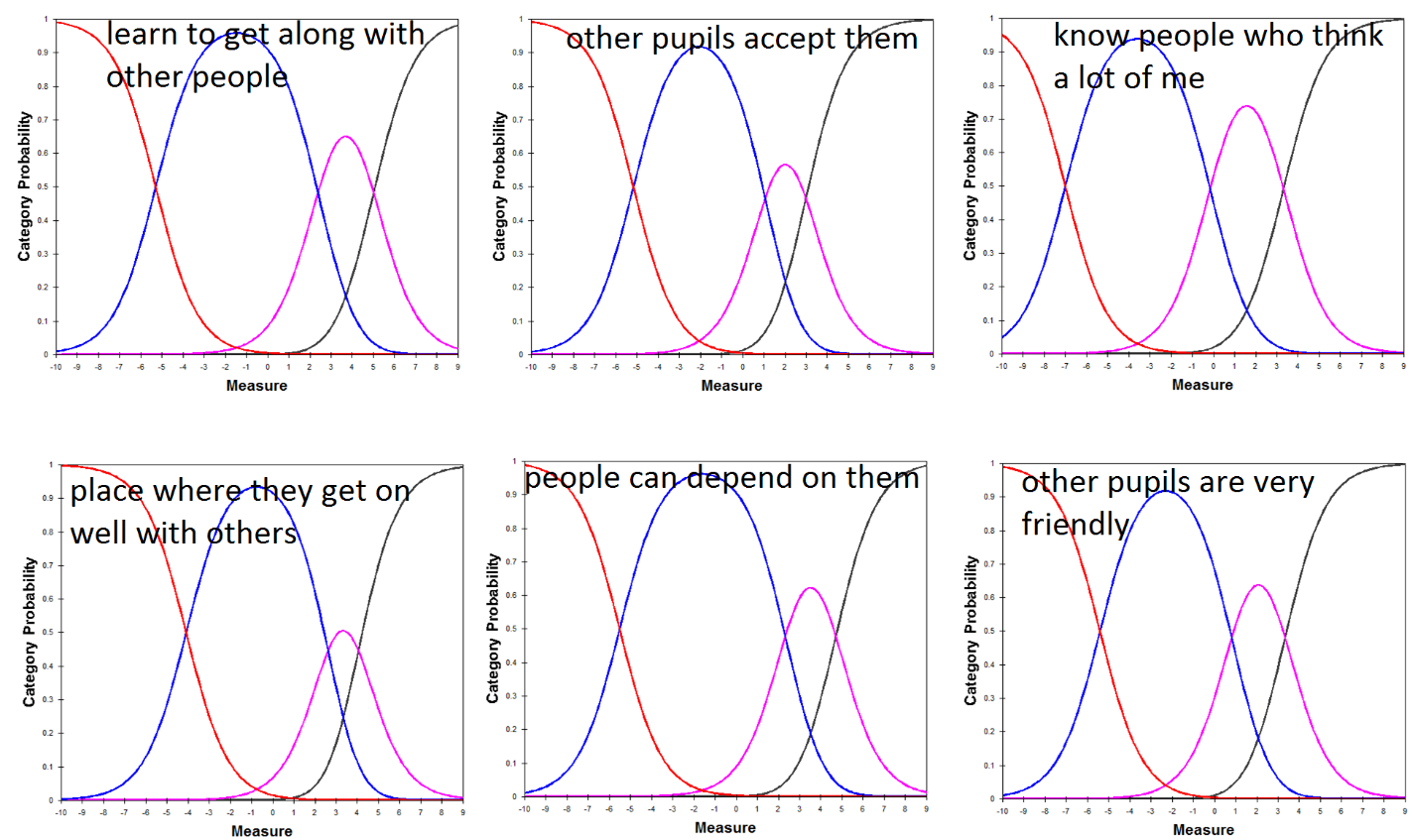
*

**Figure A5.** Person-item map of the Social Relations at School (SRaT). For the detailed interpretation of this map see the description provided in the previous section for the SI

*
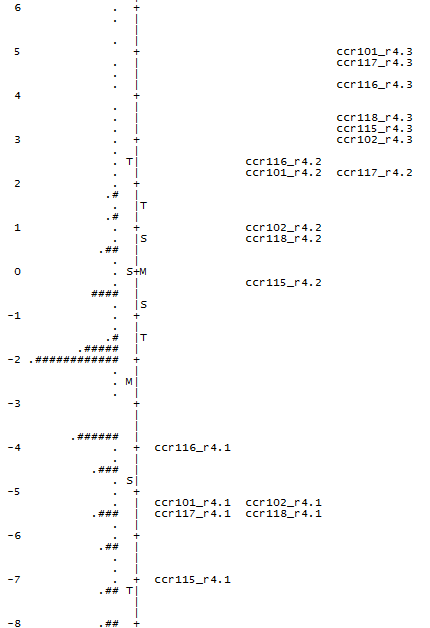
*

The following abbreviations for items are adapted:

ccr101_r4 (learn to get along );

ccr102_r4 (other pupils accept);

ccr115_r4 (know people who think a lot of me);

ccr116_r4 (place where they get on well with others);

ccr117_r4 (people can depend on them);

ccr118_r4 (other pupils very friendly).

# **Perception of Teachers**

The final scale successfully developed here was Perception of Teachers. This measure was obtained from the initial pool of 17 questions exploring several aspects of pupils’ relations with teachers at their school. The exact wording of the questions is presented in the Table A6. Respondents were asked to what extent they agree with each of them. The range of possible answers to them were running from strongly agree to strongly disagree, exactly the same way as described in previous section on perception of school (with the same coding system from 1 to 4). This is why the applied recoding schedule was also the same as the one described in the previous section. Scale was deemed that way that higher values should indicate worse perception of teachers.

After a few iteratively repeated turns removing problematic items having problems with reversed thresholds or averages (teachers will get more interest in their work than others; teachers make it clear how they should behave) or strongly misfitting ones (*If get caught breaking the rules respondent will be punished more than others*) it turned out that satisfactorily solution was obtained. It consist of 14 items with all thresholds ordered in accord with theoretical model expectations as well as misfitting indexes being within the assumed limits of acceptance (.7-1.3). All items are having point measure correlation coefficients positive and high (above.50). The overall reliability indexes are extremely high (Person reliability index=.89. ; separation index=2.83 raw score-Rasch measure correlation coefficient=.98) ensuring good level of precision of measurement and possibility of discrimination between lower and higher performers on this scale. This relatively large number of items and categories also provides a wide spread of the scale (8.67 logits). The slight problem with this scale is that there is a small gap in the middle of the Rasch continuum corresponding to the place where modal part of the sample is located. Also the highest eigenvalue from PCA on residuals is 2.0 that is borderline case. Although these two things are clearly lowering the psychometric quality of the scale the overall performance was good enough to keep this scale for further analyses.

**Table A6.** Final item set representing Perception of teachers scale. The table includes item parameters and basic indicators of item fit and discrimination (Infit, Outfit, point-measure correlation) obtained from the final model. Reliability indexes of scale are shown below the table.

| Item  **Perception of teachers** | Rasch measure  – item difficulty (SE) | | Infit MnSQ | Outfit MnSQ | Point measure  correlation | Rasch-Andrich thresholds  (50% cumulative thresholds) | | |
| --- | --- | --- | --- | --- | --- | --- | --- | --- |
| **Teachers…** |  | |  |  |  | 1 | 2 | 3 |
| Teachers take action when see anyone breaking the rules | | .49 (.03) | 1.00 | .98 | .62 | -2.95 | 1.38 | 3.05 |
| Teachers make it clear how they should behave | | .95 (.03) | .93 | .89 | .63 | -2.75 | 2.01 | 3.60 |
| Teachers don't really listen (rev.) | | -.04 (.03) | 1.00 | 1.00 | .63 | -3.52 | 1.01 | 2.38 |
| Teachers and pupils trust each other | | -1.31 (.03) | .98 | 1.00 | .66 | -5.07 | -.82 | 1.96 |
| Respondent treated unfairly by most teachers (rev.) | | .38 (.03) | 1.03 | 1.02 | .61 | -2.90 | 1.42 | 2.63 |
| Respondent make sure that any homework set is completed | | .46 (.03) | 1.14 | 1.11 | .57 | -2.95 | 1.28 | 3.05 |
| Teachers keep the class in order | | -.39 (.03) | .86 | .84 | .68 | -4.22 | .63 | 2.42 |
| People think respondent's school is good | | .09 (.03) | 1.23 | 1.22 | .57 | -2.72 | .87 | 2.13 |
| Respondent's work in lessons interest them | | -.77 (.03) | 1.00 | 1.01 | .63 | -4.78 | -.02 | 2.49 |
| Respondent's like most of their teachers | | -.43 (.03) | .92 | .90 | .65 | -4.34 | .46 | 2.59 |
| Teachers praise when they do school work well | | .18 (.03) | .88 | .85 | .67 | -3.41 | .99 | 2.96 |
| Teachers treat pupils with respect | | .26 (.03) | .81 | .76 | .71 | -3.25 | 1.17 | 2.87 |
| Teachers have given up by on some of the pupils (rev.) | | -.84 (.02) | 1.19 | 1.24 | .60 | -3.83 | -.54 | 1.85 |
| Teachers believe that all pupils can learn | | .96 (.03) | .94 | .91 | .63 | -2.58 | 2.08 | 3.38 |

Person reliability index=.89. ; separation index=2.83 raw score-Rasch measure correlation coefficient=.98

*rev*.: categories of that items were reversed (see section on recoding)

**Figure A6.** Category probabilities curves for Perception of Teachers scale obtained from the final solution

*
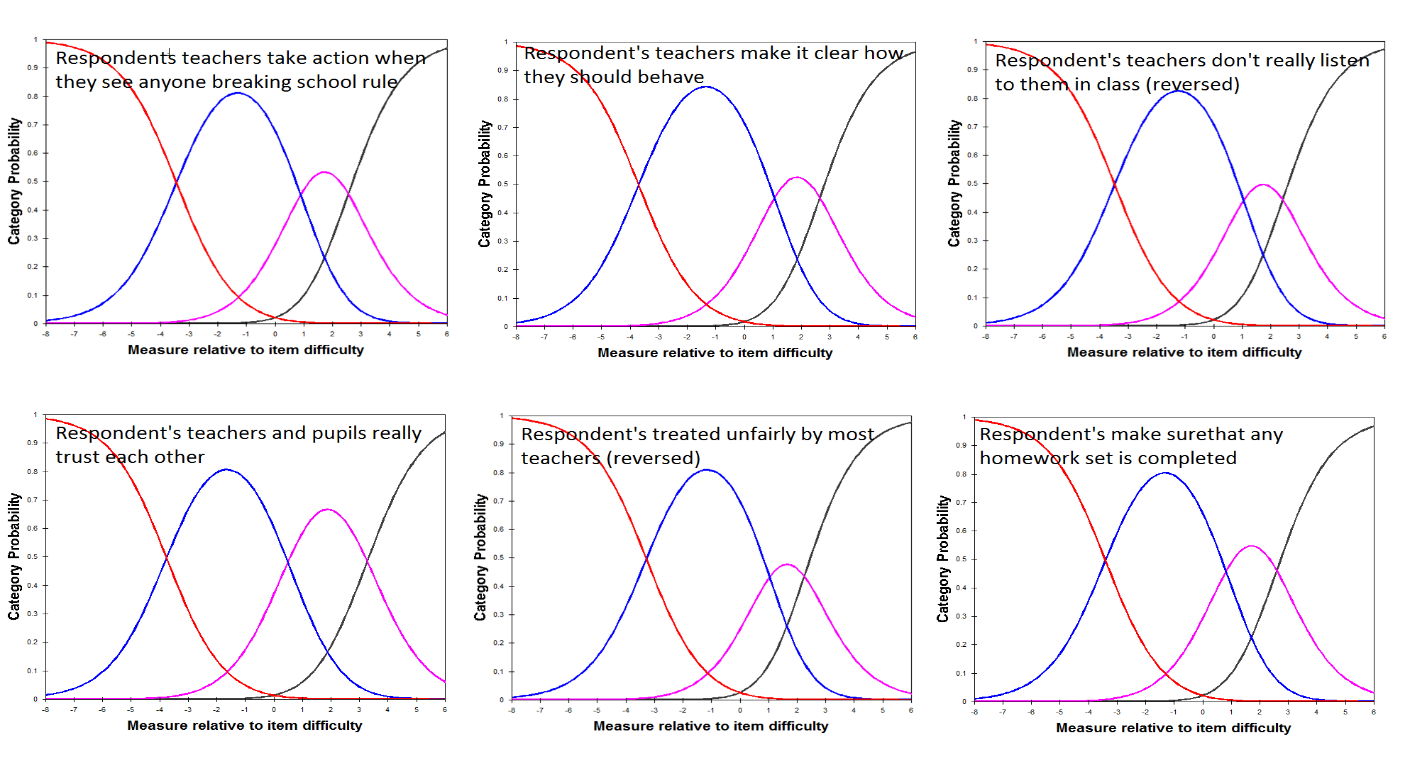
*

*
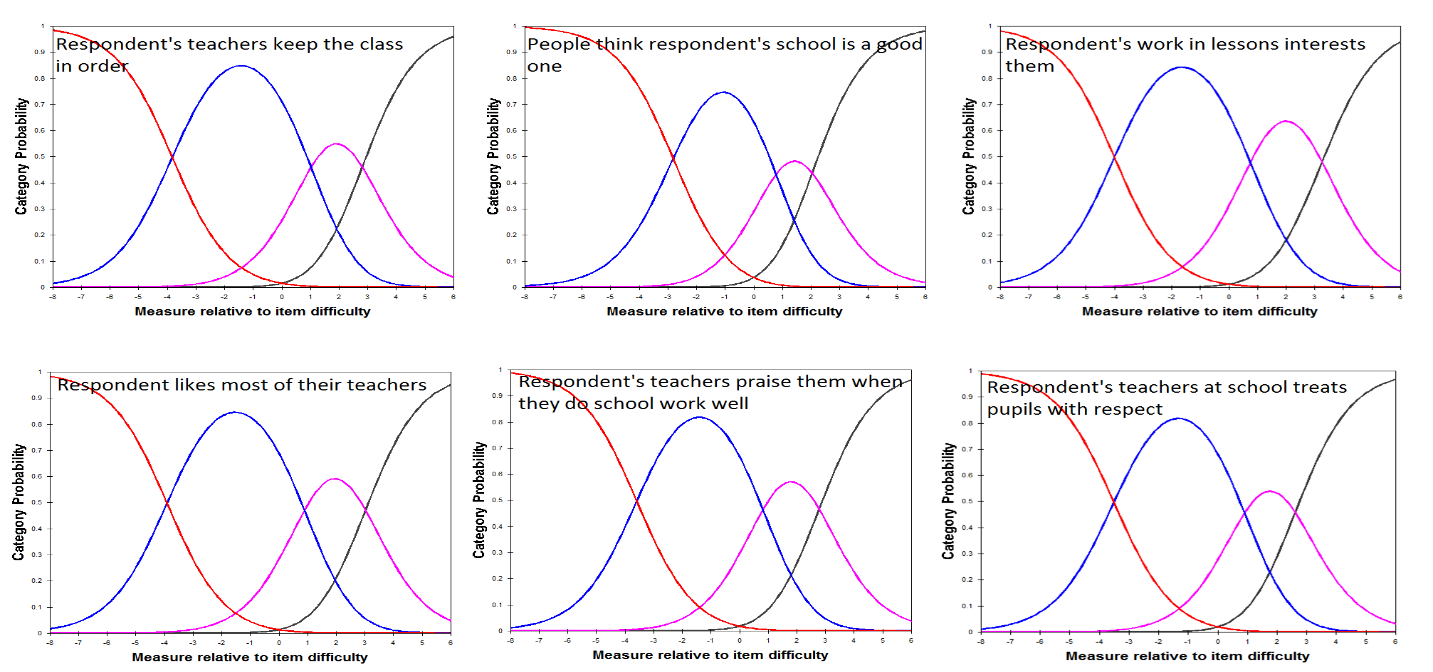

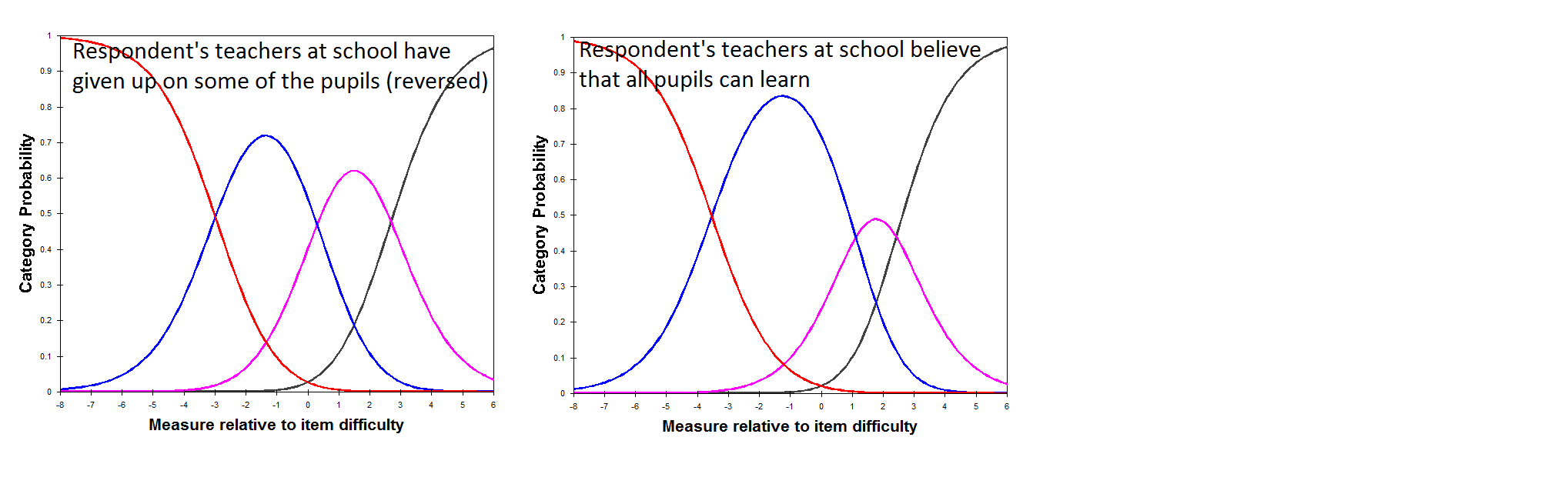
*

**Figure A7.** Person-item map of the Perception of Teachers scale (PoT). For the detailed interpretation of this map see the description provided in the previous section for the SI

*
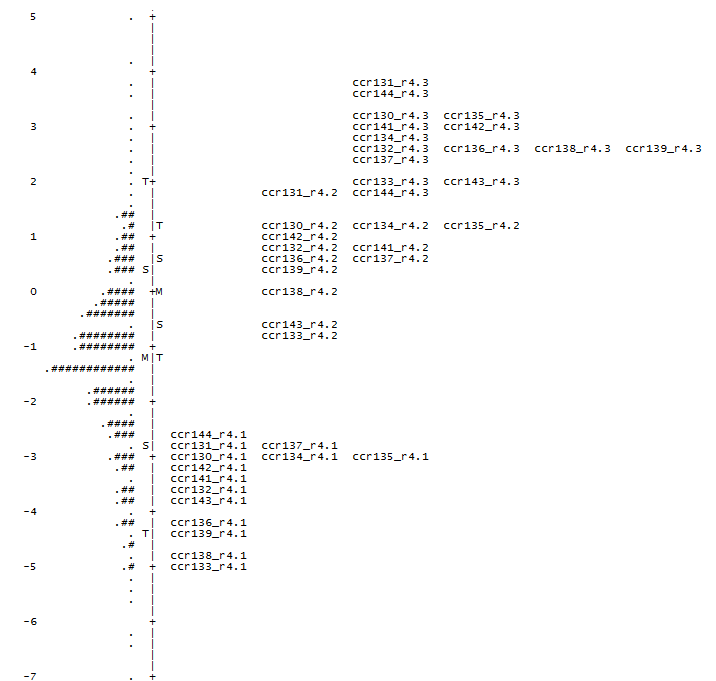
*

The following abbreviations for items are adapted: ccr130_r4 (teachers take action when see anyone breaking the rules); ccr131_r4 (teachers make it clear how they should behave); ccr132_r4 (teachers don't really listen); ccr133_r4 (teachers and pupils trust each other); ccr134_r4 (respondent treated unfairly by most teachers); ccr135_r4(respondent make sure that any homework set is completed); ccr136_r4 (teachers keep the class in order); ccr137_r4(people think respondent's school is good); ccr138_r4 (respondent's work in lessons interest them); ccr139_r4 (respondent's like most of their teachers); ccr141_r4 (teachers praise when they do school work well); ccr142_r4 (teachers treat pupils with respect); ccr143_r4 (teachers have given up by on some of the pupils); ccr144_r4 (teachers believe that all pupils can learn).

**References**

Andrich D. (1978) Rating formulation for ordered response categories. Psychometrika. ;43(3):561-573.

Andrich D. (2004) Controversy and the Rasch model: a characteristic of incompatible paradigms? Med Care. 42((1 Suppl)):7-16.

Bond TG, Fox CM. (2007) Applying The Rasch Models. 2nd ed. Mahwah, New Jersey London: Lawrence Erlbaum Associates, Publishers;

Christiansen KB, Kreiner S, Mesbah M (ed.), (2013) Rasch models in health, London, UK; Hoboken, USA: ISTE Ltd. & Wiley&Sons, Inc.

Linacre, J. M. (2008) WINSTEPS Computer Program. v.3.68.1 ; Beaverton, Oregon

Merbitz C, Morris J, Grip JC. (1989) Ordinal Scales and Foundations of Misinference. Arch Phys Med Rehabil. ;70(April):308-312.

Penta, M., Tesio, L., Arnould, C., Zancan, A., &amp; Thonnard, J.-L. (2001). The ABILHAND questionnaire as a measure of manual ability in chronic stroke patients. Rasch-based validation and relationship to upper limb impairment. Stroke, 32, 1627–1634.

Rasch G. (1960) Probabilistic models for some intelligence and attainment tests, Copenhagen: Danish Institute for Education Research

Schulz, W. and Fraillon, J. (2009). The Analysis of Measurement Equivalence in International Studies using the Rasch Mode, Paper presented to the symposium on "Rasch measurement: present, past and future" at the European Conference on Educational Research (ECER) in Vienna, 28-30 September 2009

Wright BD, Linacre JM. (1994) Reasonable mean-square fit values. Rasch Meas Trans. 8(3):370

Wright BD, Masters GN. (1982) Rating Scale Analysis. Chicago: MESA PRESS;

Wright BD, Stone MH (1979) Best Test Design, Chicago: MESA.

1. If, we substitute in equation the 1 and 0 respectively the above formula gets the following forms: $P(X_{ij}=1|\beta_{i},\delta_{j})=\frac{\mathrm{e}^{(\beta_{i}-\delta_{j})}}{1+\mathrm{e}^{^{(\beta_{i}-\delta_{j})}}}$ $P(X_{ij}=0|\beta_{i},\delta_{j})=\frac{1}{1+\mathrm{e}^{^{(\beta_{i}-\delta_{j})}}}$ [↑](#footnote-ref-1)
