# Supplementary material for: Effects of urinary incontinence on psychosocial outcomes in adolescence
Source: Eur Child Adolesc Psychiatry. 2016 Dec 10;26(6):649–58. doi: 10.1007/s00787-016-0928-0 (PMC5446552; doi:10.1007/s00787-016-0928-0)
Supplement: Supplementary file 3 — Supplementary material 3 (DOCX 89 kb) [file 787_2016_928_MOESM3_ESM.docx]

**Online Resource Part 3**

Article title: Effects of urinary incontinence on psychosocial outcomes in adolescence

# Journal name: European Child & Adolescent Psychiatry

Author names: Mariusz T Grzeda MSci^1^, Jon Heron PhD^1^, Alexander von Gontard MD PhD ^2^, Carol Joinson PhD^1^

Corresponding author: Carol Joinson, School of Social and Community Medicine, University of Bristol, Oakfield House, Oakfield Grove, Clifton, Bristol BS8 2BN, UK. Email: [Carol.Joinson@bristol.ac.uk](mailto:Carol.Joinson@bristol.ac.uk)

**Results of the sensitivity analysis re-estimating models following the exclusion of participants with urinary incontinence in adolescence**

**Table S1**. Binary outcomes – comparison of results with and without exclusion for daytime wetting and/or bedwetting in adolescence

| Depressive symptoms | Pre-exclusion^1^ (n = 5,631) | | Post-exclusion^2^ (n = 5,414) | |
| --- | --- | --- | --- | --- |
|  | Probability | SE | Probability | SE |
| Normative | 0.068 | 0.005 | 0.067 | 0.005 |
| Bedwetting alone | 0.049 | 0.010 | 0.052 | 0.011 |
| Daytime wetting alone | 0.092 | 0.024 | 0.082 | 0.024 |
| Delayed | 0.073 | 0.018 | 0.053 | 0.017 |
| Persistent wetting | 0.090 | 0.017 | 0.083 | 0.018 |
|  | Wald = 5.64, p = 0.23 | | Wald = 3.23, p = 0.52 | |

| Victimisation | Pre-exclusion^1^ (n = 5,578) | | Post-exclusion^2^ (n = 5,357) | |
| --- | --- | --- | --- | --- |
|  | Probability | SE | Probability | SE |
| Normative | 0.169 | 0.007 | 0.167 | 0.007 |
| Bedwetting alone | 0.179 | 0.017 | 0.174 | 0.018 |
| Daytime wetting alone | 0.174 | 0.033 | 0.166 | 0.034 |
| Delayed | 0.193 | 0.027 | 0.191 | 0.028 |
| Persistent wetting | 0.232 | 0.026 | 0.196 | 0.027 |
|  | Wald = 7.80, p = 0.099 | | Wald = 2.35, p = 0.67 | |

1. Pre-exclusion: sample *includes* participants with bedwetting and/or daytime wetting in adolescence.
2. Post-exclusion: sample *excludes* participants with bedwetting and/or daytime wetting in adolescence.

Estimates shown in this table are predicted probabilities of positive outcomes from the logistic regression models.

**Figure S1**. Previous comparisons depicted as point estimates with 1 SE error-bars

| Pre-exclusion | Post exclusion |
| --- | --- |
|  |  |
|  |  |

**Table S2**. Continuous outcomes – comparison of results with and without exclusion for daytime wetting and/or bedwetting in adolescence

| Self image | Pre-exclusion (n = 5,887) | | | | | | Post-exclusion (n = 5,612) | | | | | |
| --- | --- | --- | --- | --- | --- | --- | --- | --- | --- | --- | --- | --- |
|  | mean | se | Diff | SE | CI- | CI+ | mean | se | Diff | SE | CI- | CI+ |
| Normative | -0.020 | 0.019 | 0.0 ref | - | - | - | -0.029 | 0.019 | 0.0 ref | - | - | - |
| Bedwetting alone | -0.101 | 0.047 | -0.081 | 0.054 | -0.187 | 0.025 | -0.101 | 0.049 | -0.071 | 0.055 | -0.180 | 0.037 |
| Daywetting alone | 0.188 | 0.115 | 0.208 | 0.123 | -0.032 | 0.448 | 0.131 | 0.122 | 0.161 | 0.130 | -0.093 | 0.414 |
| Delayed | 0.186 | 0.068 | 0.206 | 0.069 | 0.070 | 0.341 | 0.139 | 0.071 | 0.168 | 0.072 | 0.026 | 0.310 |
| Persistent wetting | 0.011 | 0.068 | 0.030 | 0.071 | -0.108 | 0.169 | -0.104 | 0.076 | -0.075 | 0.079 | -0.229 | 0.080 |
|  |  |  |  | Wald = 16.9, p = 0.002 | | |  |  |  | Wald = 10.7, p = 0.030 | | |

| School perception | Pre-exclusion (n = 5,171) | | | | | | Post-exclusion (n = 4,936) | | | | | |
| --- | --- | --- | --- | --- | --- | --- | --- | --- | --- | --- | --- | --- |
|  | mean | se | Diff | SE | CI- | CI+ | mean | se | Diff | SE | CI- | CI+ |
| Normative | -0.052 | 0.021 | 0.000 | . |  |  | -0.056 | 0.021 | 0.0 ref | - | - | - |
| Bedwetting alone | 0.054 | 0.049 | 0.106 | 0.057 | -0.006 | 0.217 | 0.046 | 0.050 | 0.102 | 0.058 | -0.012 | 0.216 |
| Daywetting alone | 0.253 | 0.116 | 0.304 | 0.124 | 0.061 | 0.547 | 0.255 | 0.121 | 0.311 | 0.130 | 0.057 | 0.565 |
| Delayed | 0.110 | 0.073 | 0.162 | 0.075 | 0.015 | 0.309 | 0.127 | 0.076 | 0.183 | 0.078 | 0.031 | 0.335 |
| Persistent wetting | 0.003 | 0.071 | 0.055 | 0.074 | -0.090 | 0.200 | -0.033 | 0.077 | 0.023 | 0.080 | -0.134 | 0.179 |
|  |  |  |  | Wald = 14.6, p = 0.006 | | |  |  |  | Wald = 15.0, p = 0.005 | | |

| School relations | Pre-exclusion (n = 5,169) | | | | | | Post-exclusion (n = 4,934) | | | | | |
| --- | --- | --- | --- | --- | --- | --- | --- | --- | --- | --- | --- | --- |
|  | mean | se | Diff | SE | CI- | CI+ | mean | se | Diff | SE | CI- | CI+ |
| Normative | -0.067 | 0.020 | 0.0 ref | - | - | - | -0.074 | 0.020 | 0.0 ref | - | - | - |
| Bedwetting alone | 0.023 | 0.053 | 0.090 | 0.060 | -0.028 | 0.208 | 0.014 | 0.054 | 0.089 | 0.062 | -0.032 | 0.209 |
| Daywetting alone | 0.171 | 0.111 | 0.237 | 0.118 | 0.005 | 0.469 | 0.129 | 0.117 | 0.203 | 0.125 | -0.041 | 0.447 |
| Delayed | 0.211 | 0.070 | 0.277 | 0.072 | 0.136 | 0.419 | 0.193 | 0.074 | 0.267 | 0.075 | 0.119 | 0.415 |
| Persistent wetting | 0.156 | 0.072 | 0.223 | 0.075 | 0.077 | 0.369 | 0.145 | 0.079 | 0.219 | 0.082 | 0.060 | 0.379 |
|  |  |  |  | Wald = 32.8, p < 0.001 | | |  |  |  | Wald = 27.5, p < 0.001 | | |

| Teacher perception | Pre-exclusion (n = 5,162) | | | | | | Post-exclusion (n = 4,930) | | | | | |
| --- | --- | --- | --- | --- | --- | --- | --- | --- | --- | --- | --- | --- |
|  | mean | se | Diff | SE | CI- | CI+ | mean | se | Diff | SE | CI- | CI+ |
| Normative | -0.030 | 0.021 | 0.0 ref | - | - | - | -0.035 | 0.021 | 0.0 ref | - | - | - |
| Bedwetting alone | 0.043 | 0.051 | 0.073 | 0.059 | -0.043 | 0.188 | 0.031 | 0.052 | 0.065 | 0.060 | -0.052 | 0.183 |
| Daywetting alone | 0.131 | 0.116 | 0.161 | 0.124 | -0.082 | 0.404 | 0.141 | 0.121 | 0.175 | 0.129 | -0.077 | 0.428 |
| Delayed | 0.058 | 0.074 | 0.088 | 0.076 | -0.061 | 0.236 | 0.057 | 0.076 | 0.092 | 0.077 | -0.059 | 0.243 |
| Persistent wetting | -0.006 | 0.069 | 0.024 | 0.072 | -0.118 | 0.166 | 0.037 | 0.077 | 0.071 | 0.080 | -0.084 | 0.227 |
|  |  |  |  | Wald = 4.67, p = 0.32 | | |  |  |  | Wald = 5.30, p = 0.26 | | |

Estimates are means (on the standardized Rasch scales) and mean differences relative to the normative class

**Figure S2**. Previous comparisons depicted as point estimates with 1 SE error-bars

| Pre-exclusion | Post exclusion |
| --- | --- |
|  |  |
|  |  |
|  |  |
|  |  |
